# Optoelectronics of Single Mixed Dislocations in Van Der Waals Core‐Shell Nanowires

**DOI:** 10.1002/smll.74258

**Published:** 2026-06-19

**Authors:** Eli Sutter, Peter Sutter

**Affiliations:** ^1^ Department of Mechanical & Materials Engineering University of Nebraska‐Lincoln Lincoln Nebraska USA; ^2^ Department of Electrical & Computer Engineering University of Nebraska‐Lincoln Lincoln Nebraska USA

**Keywords:** cathodoluminescence, dislocations, light emission, nanowires, recombination, van der Waals crystals, vapor‐liquid‐solid growth

## Abstract

While dislocations are traditionally perceived as detrimental to electronic materials, recent theories predicted emerging functional properties associated with dislocations. To identify and ultimately harness such functionality, approaches are needed for embedding single dislocations with tunable geometry (edge/screw ratio) in small‐volume host crystals. Here, we address this challenge by mixing screw‐ and edge‐dislocations in Ge_1‐x_Sn_x_S layered (van der Waals) core‐shell nanowires. Phase separation during vapor‐liquid‐solid growth yields the required core‐shell structure, and the growth process also produces a homojunction between a defect‐free layered segment near the tip and a base segment containing a single mixed (helical) dislocation. Nanometer‐scale cathodoluminescence (CL) spectroscopy sheds light on the effects of single helical dislocations on optoelectronics. CL shows that the luminescence intensity depends on the distance from the dislocation line. Efficient radiative recombination is found for the pristine material near the nanowire tip whereas the dislocated part of the nanowires shows a sharp reduction of the spontaneous emission quantum efficiency, attributed to the edge component of the helical dislocation. The results demonstrate control over the geometry of single mixed dislocations and the ability of probing their effect on functional properties, important steps toward the ultimate use of dislocations as active elements in devices.

## Graphical abstract

Ge_1‐x_Sn_x_S van der Waals core‐shell nanowires can host single helical dislocations whose character is tunable from a pure screw to a majority edge dislocation via the core‐shell lattice mismatch. Cathodoluminescence shows profound effects once an edge component is introduced, notably a lowering of the spontaneous emission quantum efficiency due to the onset of deep‐level nonradiative recombination.

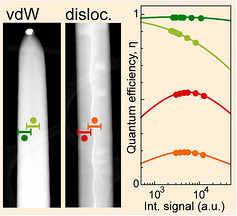

## 1 Introduction

Dislocations are extended crystal defects that can affect the properties of materials and play a crucial role in a wide range of natural and technological processes. Spiral growth around screw dislocations, for example, facilitates crystal growth by enabling continuous materials incorporation without nucleation [1]. Dislocations also define the mechanical properties of metals and alloys. In semiconductors, significant efforts have been dedicated to the elimination of dislocations as they cause charge carrier scattering and decrease mobility in devices. Recent research, however, has indicated that dislocations need not always be detrimental but, on the contrary, may be capable of endowing materials with new functionalities. Theoretically predicted emerging electronic properties include dislocation spin‐orbit coupling generating highly coherent spin textures [2], Majorana bound states [3], chiral anomalies with emergent magnetic flux in Dirac semimetals [4, 5], probes of monopole charge in multi‐Weyl semimetals [6], and low‐dimensional gapless modes in topological insulators [7]. Recent theoretical work has established a unified framework in which dislocations are treated as quantized quasiparticles (*‘dislons’*) analogous to phonons [8, 9]. The quantum theory of dislocations predicts functional properties such as electronic effects, including an oscillatory electron energy near a dislocation core [10], effects on phonons [11], dislocation‐induced superconductivity [12], as well as topological and quantum phase transitions. In 2D/layered crystals, dislocations—and more generally defect and strain engineering [13, 14] – are being investigated for quantum information processing, for example, by creating and controlling single photon emitters in materials including transition metal dichalcogenides [15, 16] and hexagonal boron nitride [17].

Electrical and optical investigations established that dislocation cores can act as highly conductive carrier channels, modify the electronic properties of materials [18], and support high photoconductivity [19] and the bulk photovoltaic effect [20]. The majority of these prior investigations involved the creation of extended dislocation networks [21, 22] or a high density of dislocations [23]. In order to harness the properties of dislocations, studies of isolated dislocations are needed, ideally with controlled placement of single defects in small volumes to maximize their effects. Realizing such control is extremely challenging [18]. Therefore, previous studies of the effects of single dislocations relied on spatially resolved measurements, such as electron‐beam induced current (EBIC) and cathodoluminescence (CL) to target individual defects that were statistically distributed among large ensembles, usually with no or limited control over the dislocation type. STEM‐EBIC and STEM‐CL on misfit dislocations in Ga_1‐x_Al_x_As_1‐y_P_y_ heterostructures, for example, generally showed enhanced nonradiative recombination near the dislocation lines but also identified a type of ‘clean’ dislocation with a heavily reconstructed core (and hence absence of dangling bonds) via its high carrier collection efficiency in EBIC and lack of luminescence quenching in CL [24]. In diamond, different types of dislocations, including edge‐, screw‐, and mixed (60°) dislocations, were found to show in‐gap radiative transitions, but no systematic correlation of the luminescence with dislocation character was found [25]. Low‐temperature STEM‐CL on ZnSe and InP, containing ensembles of dislocations formed during growth, showed differences in the spectra, notably band‐edge luminescence away from and defect emission near dislocations [26]. Recent work used CL in scanning electron microscopy (SEM‐CL) to investigate the effects of single threading dislocations on drift, diffusion, and recombination of excitons in GaN [27, 28].

**FIGURE 1:**
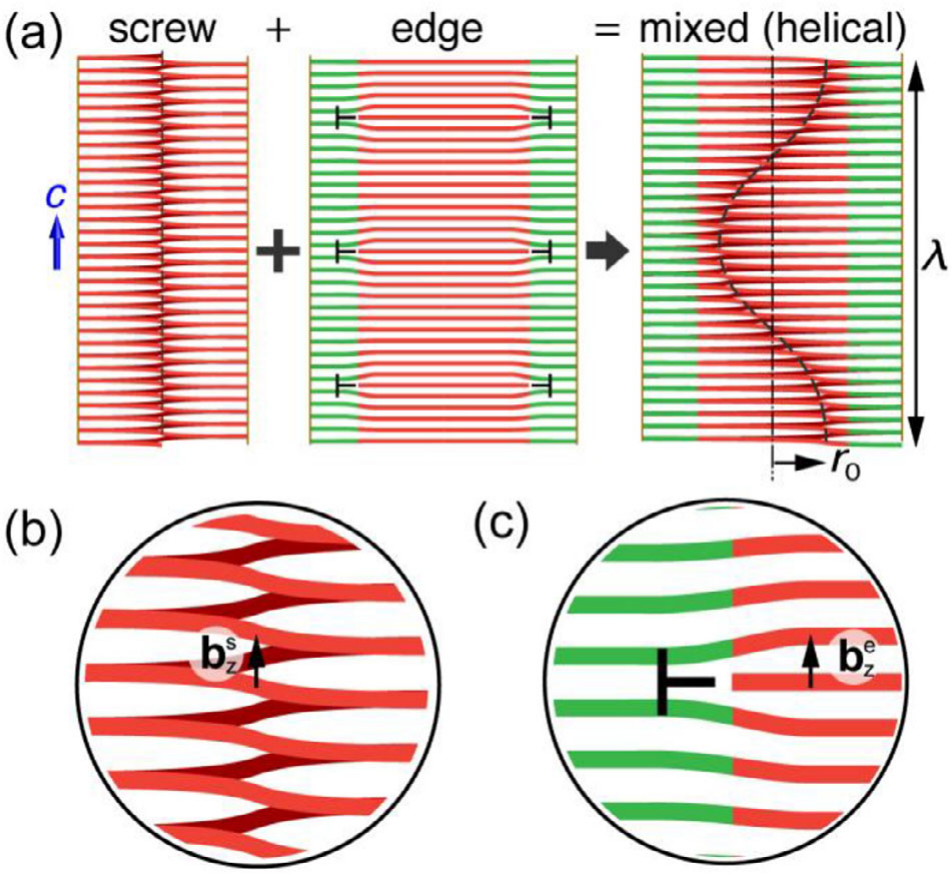
Dislocations in van der Waals nanowires. (a) Schematic cross‐section of a layered nanowire showing how combining an axial screw dislocation with *c*‐axis misfit (edge) dislocations in a core‐shell structure produces van der Waals nanowires carrying single helical dislocations with mixed screw‐ and edge character. (b) Burgers vector of a pure screw dislocation in a layered nanowire. (c) Burgers vector of a pure edge dislocation in a layered core‐shell nanowire.

We recently demonstrated that it is possible to isolate individual dislocations with tunable properties in nanowires of layered crystals [29]. Generally, there are two pure forms, edge and screw dislocations, which are characterized by a lattice distortion (measured by the Burgers vector, **b**) perpendicular and parallel to the dislocation line direction, respectively. In real materials, pure as well as mixed dislocations combining screw and edge character with multiple symmetry‐allowed configurations are frequently present. The layer structure of van der Waals crystals helps reduce the number of possible dislocation configurations, thus facilitating the creation and manipulation of single dislocations. Layered van der Waals crystals can host pure as well as mixed dislocations. Pure screw dislocations in layered crystals provide avenues for synthesizing van der Waals stacks with controlled interlayer twist [30, 31] and tend to be benign in optoelectronics [29]. Nanowires of the layered group IV monochalcogenide semiconductor GeS have been shown to be suitable hosts for individual dislocations. While homogeneous GeS nanowires carry single screw dislocations [32], the synthesis of radial nanowire heterostructures (GeS‐Ge_1‐x_Sn_x_S monochalcogenide core‐shell nanowires) can transform the defect into a mixed (helical) dislocation whose edge/screw ratio is tunable via the lattice mismatch between core and shell (Figure 1; Figure S1) [29].

Here, we use this capability to investigate the optoelectronic properties of single mixed dislocations with variable edge/screw ratio, embedded in the small volume of a nanowire host. We demonstrate the controlled synthesis of alloy core‐shell nanowires containing individual mixed helical dislocations in a vapor‐liquid‐solid (VLS) growth process using SnS and GeS precursors. In addition, we employ a special thermal treatment near the end of the growth process to induce the transition from dislocated to a defect‐free segment within the same nanowire, thus creating a homojunction between dislocated and pristine portions of the van der Waals wire. Transmission electron microscopy (TEM) and electron diffraction in combination with energy dispersive X‐ray spectroscopy are used to determine the structure and composition of the core‐shell nanowires, along with the edge/screw ratio of the helical dislocations. Cathodoluminescence spectroscopy in scanning transmission electron microscopy (STEM‐CL) is used to probe the optoelectronic properties of nanowires containing individual helical dislocations, contrasting light emission from defect‐free and dislocated segments of the same nanowires and comparing light emission due to radiative recombination as a function of distance from the dislocation line. The work contrasts with prior studies (discussed above) in two key aspects: (i) nanowires carrying single dislocations create a platform where the line defect is embedded in a small volume host, suitable for identifying not only its effects on optoelectronics (as discussed here) but also other properties; and (ii) the character of the defect is continuously variable between a pure screw‐ and nearly pure edge dislocation via the core‐shell lattice mismatch, without significant other changes to the host crystal. Finally, the presence of dislocated and defect‐free segments in the same nanowire provides a perfect single crystal reference with identical composition, surface effects, etc., as the part containing the dislocation.

## 2 Results and Discussion

### 2.1 Single Dislocations in Layered Ge_1‐x_Sn_x_S Core‐Shell Nanowires

**FIGURE 2:**
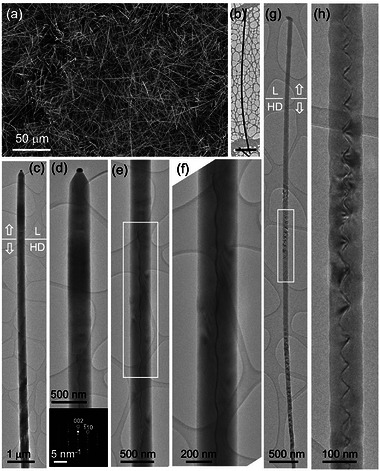
Van der Waals nanowires with single mixed (helical) dislocations. (a) SEM image of the forest of nanowires with helical dislocations on the Au/Si(100) growth substrate. (b) TEM image of a nanowire transferred to a carbon grid. Scale bar: 5 µm. (c) TEM image of the nanowire, showing the segment containing a single helical dislocation (HD) transitioning to a defect‐free layered structure (L) near the tip. (d) High‐magnification TEM image of the nanowire's defect‐free part close to the tip. Inset: Electron diffraction of the nanowire along the [110] zone axis, showing the *c*‐axis ([001] direction) of the crystal aligned with the symmetry axis of the wire. (e) Closeup TEM image showing the transition between the nanowire with a helical dislocation and the non‐dislocated segment near the tip. (f) High‐resolution TEM image of the helical dislocation near its exit point from the wire (area marked in (e)). (g) TEM image of another nanowire containing a helical dislocation. (h) Detail showing the helical dislocation (area marked in (g)).

GeS nanowires synthesized by VLS growth using different catalysts (Au, Bi) [30, 31] on Si substrates invariably contain individual axial screw dislocations that guide the facile growth via continuous attachment, that is, without requiring any new layer nucleation. Growth on other substrates, for example, SnSe van der Waals supports, can lead to the suppression of the dislocation and give rise to defect‐free monocrystalline nanowires [33]. Introduction of SnS vapor during growth can affect the stacking of the layered crystal [34, 35], and under certain growth conditions [29] leads to the formation of core‐shell nanowires (see Methods for details). VLS growth from combined SnS and GeS precursors over Au catalysts on Si substrates typically produces large quantities of nanowires (Figure 2a). The large majority of the nanowires are single‐crystalline (Figure 2c) and host individual axial helical dislocations (Figure 2e–h). Pure screw dislocations are distinguishable by their straight dislocation line [30]. Deviations from the straight line indicate the introduction of an edge component of the Burgers vector that transforms the screws into mixed helical dislocations (Figure 1a). The characteristic meandering (sinusoidal) contrast in electron microscopy corresponds to the projection of a helical dislocation line viewed perpendicular to the axis of the helix. Figure 2c,g) shows characteristic TEM images of two nanowires hosting helical dislocations grown under different conditions. Higher magnification TEM images of the helical dislocations show small (Figure 2f) as well as significant (Figure 2h) deviations from the straight line typical of pure screw dislocations found in homogeneous GeS nanowires. These differences between helical dislocations are due to their distinct edge/screw ratios [29], controlled by the composition and Sn content (see below) of the nanostructures defined by the growth conditions.

In addition, the TEM images show that the dislocations do not persist up to the very tip of the nanowires, that is, the nanowires contain homojunctions between a layered defect‐free (L) structure near the tips and segments with helical dislocations (HD) over the remainder of the nanowire to the base. TEM images and electron diffraction patterns near the nanowire tips (Figure 1d) show their single‐crystalline structure with [001] layering direction along the nanowire axis. For our growth conditions, this defect‐free layered structure is typically found in segments with lengths of ∼2.6 µm at the tips, as seen for both nanowires in Figure 2c,g. The junctions are obtained spontaneously by triggering the expulsion of the dislocations during cooling near the end of the growth process. A similar ejection of dislocations has been demonstrated in pure GeS nanowires, where in the final segment near the tip defect‐free layered GeS was obtained. Prior work has linked this elimination of the dislocation from the wires to the injection of excess vacancies, mediating the climb of the dislocation to the surface with the onset of cooling near the end of the growth. The final (non‐dislocated) segment of the nanowire then grows through a regular VLS process [36]. The formation of homojunctions between dislocated and non‐dislocated (defect‐free) segments in the nanowires grown from mixed SnS‐GeS vapors allows us to probe the optoelectronic effects of single helical dislocations in direct comparison to the non‐dislocated single‐crystalline material within the same nanowire.

### 2.2 Energy Dispersive X‐Ray Spectroscopy of the Core‐shell Structure

**FIGURE 3:**
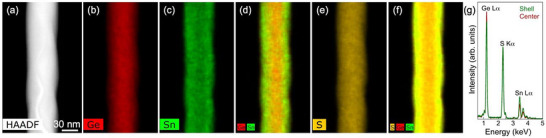
Core‐shell structure of the nanowires with helical dislocations. (a) HAADF‐STEM image of a typical nanowire with helical dislocation. (b)–(f). EDS elemental and combined maps of the distribution of germanium, tin and sulfur in the nanowire. (g) EDS spectra measured at the center (red curve) and at the periphery shell (green curve) of the nanowires. The scale marker in (a) applies to all panels.

Energy dispersive X‐ray spectroscopy (EDS) elemental maps and spectra show the presence of Ge, Sn, and S in the nanowires, i.e., the wires consist of Ge_1‐x_Sn_x_S compounds (Figure 3; Figures S2 and S3). Elemental (Figure 3b,c) and overlay (Figure 3d) maps show that the distribution of the cations (Ge and Sn) is not homogeneous. Instead, the nanowires consist of a germanium‐rich core and a tin‐enriched near‐surface region. Sulfur elemental and overlay maps, on the other hand, show uniform chalcogen content (Figure 3e,f), consistent with a constant anion to cation ratio across the nanowires. Thus, the alloy nanowires consist of a SnS‐rich shell surrounding a GeS‐rich core. EDS spectra across the wires confirm the higher Sn content in the shell compared to the core (Figure 3g). A precise quantification of the core composition is challenging since the exciting electron beam traverses both shell and core, so that both parts contribute to the measured (apparent) composition. Based on the measured composition of the Sn‐rich near‐surface region (shell: 35 at. % Ge; 15 at. % Sn) and the composition in the central region (core + shell: 40 at. % Ge; 10 at. % Sn), along with the observed core diameter (∼40 nm) and shell thickness (∼25 nm, measured from Figure 3d), the actual core composition can be estimated to 46 at. % Ge and 4 at. % Sn (Figure S4). This implies that the core consists of a GeS‐rich alloy (Ge_0.92_Sn_0.08_S) while the shell contains a considerable amount of Sn (Ge_0.7_Sn_0.3_S).

Importantly, the EDS results show that Ge_1‐x_Sn_x_S alloy nanowires with different compositions invariably crystallize as core‐shell nanostructures. The phase separation and formation of a core‐shell structure can occur either immediately upon incorporation from the molten VLS catalyst or shortly thereafter via solid‐state diffusion. The transformation of the twisted GeS nanowires containing pure screw dislocations into core‐shell wires upon introduction of SnS holds the key to generating tunable mixed dislocations (see Figure 1). Lattice mismatch between the core and the shell in the axial direction (i.e., in the *c*‐axis (interlayer) spacing) in Ge_1‐x_Sn_x_S core‐shell wires would normally be relaxed by edge dislocations. However, as the pure GeS nanowires already contain a screw dislocation, the screw and the array of misfit edge dislocations combine into a single helical dislocation whose Burgers vector contains both screw and edge components (Figures 1a and 2f,h). The edge/screw ratio is determined by the core‐shell misfit, which in turn is selected via the amount of Sn in the nanowire shell. That is, the core‐shell lattice mismatch directly affects the geometry of the dislocation line (and hence the ratio between screw‐ and edge character of the mixed dislocation). When the Sn content is low (overall composition Ge_0.92_Sn_0.08_S for the dislocation in Figure S2), the resulting helical dislocations deviate only slightly from the straight line characteristic of pure screw dislocations. Increasing the Sn content (and thus the core‐shell mismatch) leads to pronounced spirals (e.g., Figure 3; overall composition Ge_0.74_Sn_0.26_S). Note that to distinguish between core‐shell nanowires with different helical (mixed) dislocations below, we will refer to the overall composition determined from the quantification of the spectra summed over the entire nanostructure. Generally, the higher the overall Sn content the larger the edge component of the helical dislocation in the nanostructure.

### 2.3 Mixed Helical Dislocations With Tunable Edge/Screw Ratio

**FIGURE 4:**
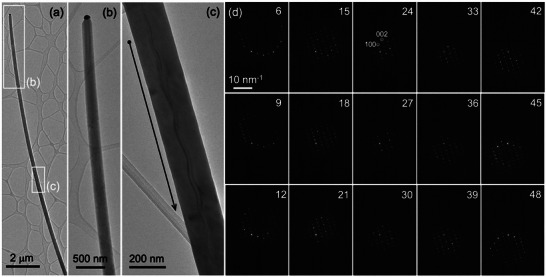
Twist in van der Waals nanowires with single helical dislocations. (a) TEM image of a nanowire transferred to a carbon grid. (b) TEM image of the nanowire near the tip, where it has a defect‐free layered structure. (c) TEM image showing the segment of the nanowire that contains a single helical dislocation, where a sequence of 50 nanobeam electron diffraction patterns was measured (black line; start at the dot, step of 20 nm). (d) Selected nanobeam electron diffraction patterns from the sequence obtained along the black line in (c) showing progressive twist of the in‐plane (a,b) crystal axes along the nanowire as it rotates onto and away from the [010] zone axis. Numbers in the different panels indicate at which step the diffraction patterns were recorded. The Eshelby twist of the helix is determined to be 4.4°/µm.

Diffraction analysis of the Ge_1‐x_Sn_x_S core‐shell nanowires demonstrates a twisted structure in the presence of helical dislocations (Figure 4). Nanobeam electron diffraction patterns recorded along the nanowires show the lattice rotation of the (*a*, *b*) crystal axes associated with Eshelby twist [37] (Figure 4d) due to the screw component of the helix with Burgers vectors $b_{z}^{s}$ (see Figure 1b), usually given in multiples of the *c*‐axis unit cell size (1.04 nm, comprising an AB‐stacked bilayer) [30, 31, 36]. The wire in Figure 4a (diameter 2*R* = 228 nm, helix radius *r*_0_ = 10 nm, helix pitch λ = 345 nm), for example, shows a twist of ∼4.4°µm^−1^. The formation of the helical line involves the introduction of an edge component to the overall Burgers vector. In a van der Waals crystal, the Burgers vector of this edge component, $b_{z}^{e}$, which is parallel to the nanowire axis (i.e., the *c*‐axis, see Figure 1c), is also quantized in multiples of the interlayer spacing. For helical dislocations in layered crystals, the edge/screw ratio of the Burgers vector is given by $|b_{z}^{e}\left|/\right|b_{z}^{s}|=\tan\theta$, where *θ* is the angle between the overall Burgers vector b_z_ (parallel to the nanowire axis) and the helical dislocation line (Figure S1). In core‐shell wires with low shell Sn‐content (i.e., small core‐shell mismatch; Figure 4), *r*_0_ and *θ* remain small, that is, the dislocation line stays close to the straight line of the incipient screw dislocation. The measured (*r*_0_, *λ*) in the sample of Figure 4 concurs with the measured angle *θ* ≈ 8° between the nanowire axis and the dislocation line. Hence, the helix adopts a fixed $b_{z}^{e}/b_{z}^{s}\approx 1/7.1$. Increasing the core‐shell mismatch through a higher Sn content of the shell (Figure S3), yields *θ* ≈ 45°, that is, brings the edge/screw ratio to ∼1/1.

### 2.4 Optoelectronics of Single Mixed Dislocations in Ge_1‐x_Sn_x_S Core‐Shell Nanowires

The realization of helical dislocations with tunable edge/screw ratio allows us to investigate the effects of single dislocations on light emission and optoelectronic properties. In addition, the homojunction connecting dislocated and defect‐free segments in the same nanowire provides a reference for the optoelectronic properties of a defect‐free wire with identical core‐shell composition and structure. In 3D semiconductors, screw dislocations were found to be relatively benign in carrier transport and recombination [38] while edge dislocations give rise to deep‐level nonradiative recombination centers in the gap [39, 40] and have a complicated recombination behavior [21].

We used nanoscale optoelectronic measurements, specifically single‐nanowire CL excited by the focused electron beam in STEM, to track the electronic structure modifications due to single helical dislocations. CL line scans across single nanowires were used to probe radiative recombination in the core and s regions of wires with and without helical dislocations. In these experiments, the focal diameter of the exciting electron beam is about 1 nm. By positioning the beam at different distances from a dislocation line (embedded here in nanowires with diameters of ∼100 nm), the effects of the dislocation on recombination and light emission can be examined. It is important to note, however, that the excitation affects a larger volume traversed by the beam along its propagation direction. This unavoidably causes some mixing of signals from the defect and the adjacent regions of the nanowire.

**FIGURE 5:**
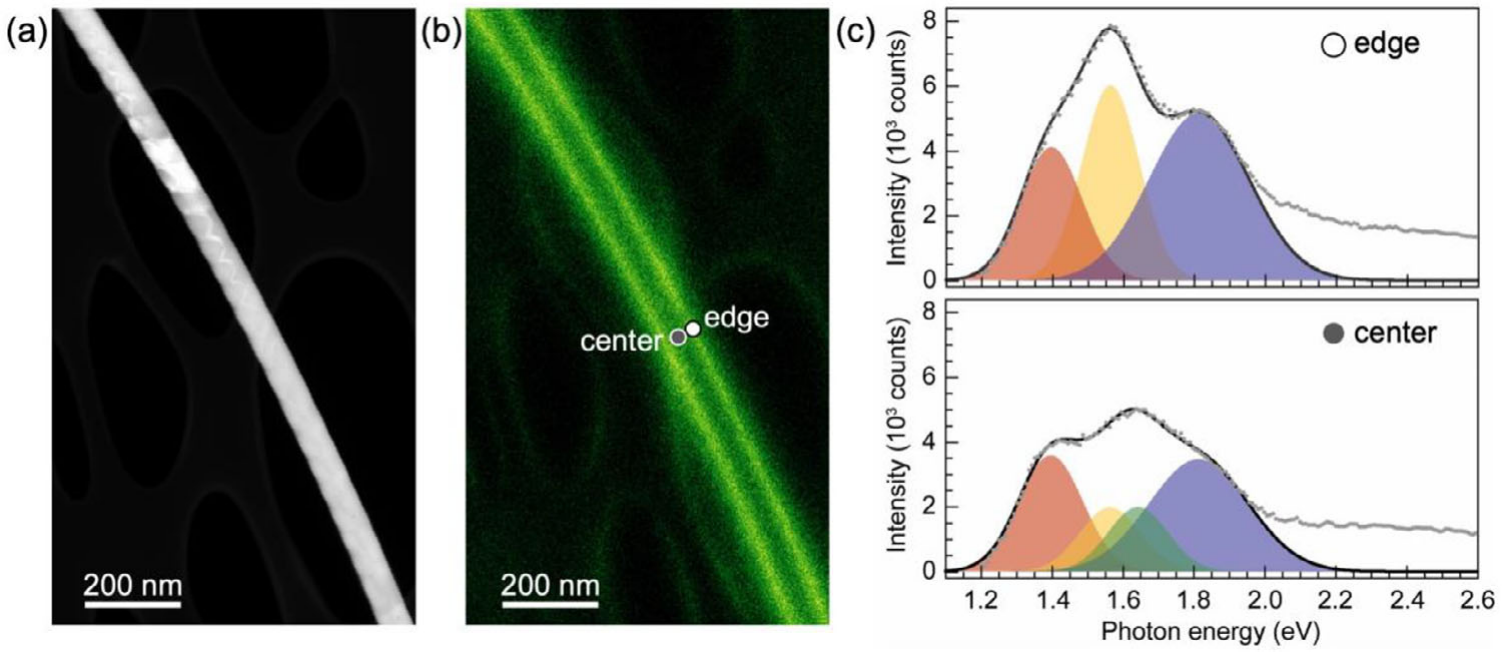
Characteristic CL spectra obtained by electron‐beam excitation at the center and edge of dislocated nanowires. (a) STEM image of a nanowire carrying a single helical dislocation. (b) Panchromatic CL intensity map integrating wavelengths between 400 nm and 1000 nm. (c) CL spectra obtained with excitation at the edge and center of the dislocated nanowire. Shaded regions indicate the components of a multi‐Gaussian line shape analysis of the experimental spectra.

Figure 5 illustrates typical data obtained on a nanowire carrying a single helical dislocation with a considerable edge/screw ratio (∼1/1; *θ* ≈ 45°). Figure 5a shows a STEM image of the nanowire, in which the helical dislocation can be seen as a meandering line with bright contrast. A panchromatic CL map of the same wire shows the highest emitted intensity near the edge and lower emission from the center (Figure 5b). The spectral line shape of the emitted light is analyzed in Figure 5c. The most obvious difference is a ∼50% lower peak intensity in the center compared to the edge, as seen also in the panchromatic map. The spectra can be analyzed further by multi‐component fitting. In the edge region, the low‐energy emission at photon energies below 1.8 eV can be deconvoluted into 3 Gaussian components: Two narrow peaks centered at 1.40 eV (red) and 1.56 eV (yellow), respectively, and a wider emission centered at 1.81 eV (blue). Fitting using the same Gaussian components does not fully reproduce the spectrum from the central region, indicating two changes to the spectral line shape in the vicinity of the helical dislocation. First, an additional spectral component, centered at 1.64 eV (green), is required to fit the modified peak shape; and secondly, the intensity of the dominant emission peak near the edge (centered at 1.56 eV, yellow) is strongly suppressed in the center, that is, close to the line of the helical dislocation. CL linescans parallel to the nanowires confirm that the spectra are highly reproducible, that is, independent of the position along a wire (Figure S5).

**FIGURE 6:**
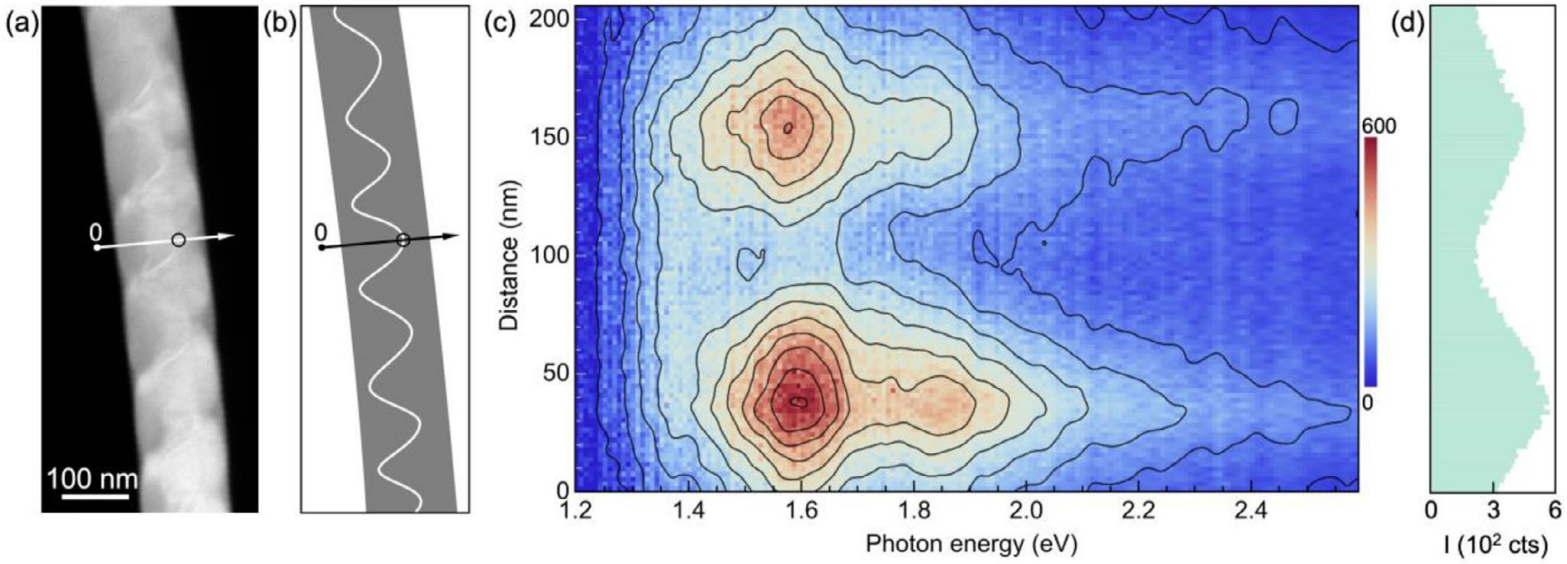
Differences in emitted light intensity close to and far from the helical dislocation line. (a) STEM image of a nanowire carrying a single helical dislocation. (b) Schematic indicating the position of the helical dislocation line (white trace) relative to a STEM‐CL linescan (arrow). (c) Hyperspectral CL data obtained along the arrow shown in panels (a) and (b). (d) Intensity profile of the main peak centered just below 1.6 eV photon energy.

Figure 6 confirms the locally altered radiative recombination near the line of the helical dislocation. Figure 6a shows a STEM image of a segment of a nanowire carrying a single helical dislocation. A CL linescan was measured across the wire, as indicated in Figure 6a,b. Note that the linescan first traverses material far from the dislocation until crossing the dislocation line at about 2/3 of the overall distance. The hyperspectral data along this line are summarized in Figure 6c. The spectra conform to the behavior seen in Figure 5, that is, near the edge show high emitted intensity with a peak at ∼1.56 eV and a broader tail toward higher photon energy, whereas the core emits at substantially lower intensity and with a slightly modified spectral content (see Figure 5c). Note, however, that the band‐edge emission at 1.56 eV remains the main emission also near the dislocation line, that is, lower‐energy radiative transitions between defect (gap) states [26, 41] are not apparent near the dislocation line. Comparing the two edge regions on either side of the nanowire, we find that the intensity is ∼30% higher on the side away from the dislocation than near the line of the helical dislocation (Figure 6c). Measurements on other nanowires show the same effect, that is, confirm the reduction of the emitted light intensity near the line of the single dislocation (see Figure S6).

Prior work showed that pure screw dislocations are benign optoelectronically in layered GeS nanowires [29]. To quantify the effects of gap states due to the introduction of the edge component of single helical dislocations, we analyzed the competition between radiative and nonradiative recombination in non‐dislocated and dislocated Ge_1‐x_Sn_x_S core‐shell segments of the same nanowire via the spontaneous emission quantum efficiency probed by electron beam current‐dependent CL [34].

**FIGURE 7:**
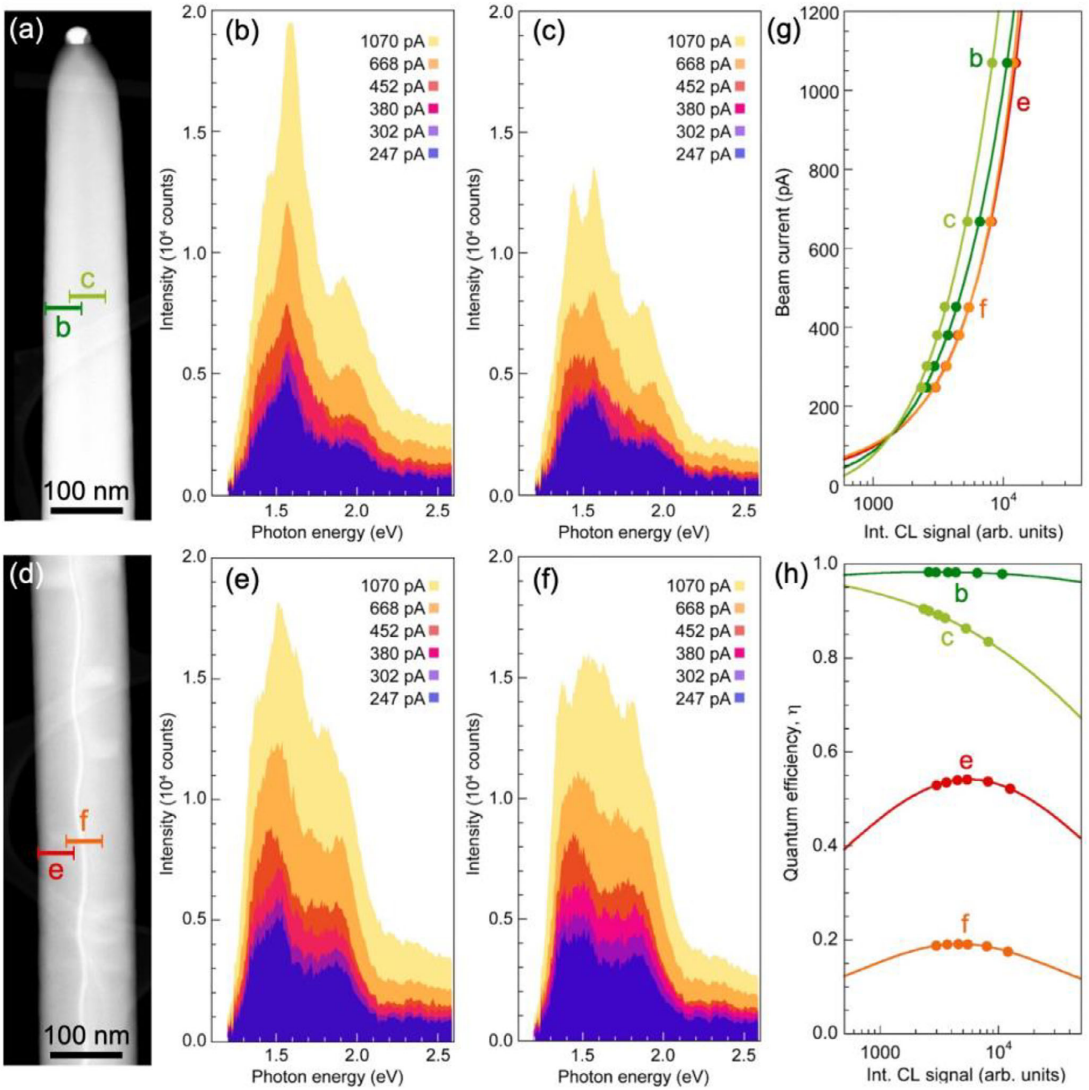
Spontaneous emission quantum efficiency of defect‐free and dislocated segments in the same core‐shell nanowire. (a) HAADF‐STEM image of the defect‐free core‐shell nanowire segment near the tip. (b,c) Beam current‐dependent CL spectra (for electron beam currents between 247 and 1070 pA) of the defect‐free nanowire segment, measured at the edge (b) and in the center (c) of the wire, respectively (integrating across regions shown in (a)). (d) HAADF‐STEM image of the segment of the same core‐shell nanowire carrying a single helical dislocation with edge/screw ratio of $b_{z}^{e}/b_{z}^{s}\approx 1/7.1$ (*θ* ≈ 8°). (e,f) Beam current‐dependent CL spectra of the nanowire segment carrying a helical dislocation, measured at the edge (e) and in the center (f) of the wire, respectively (integrating across regions shown in (d)). (g) Illustration of fits of beam current versus integrated CL signal, used to extract parameters *A*_CL_ (Shockley‐Read‐Hall nonradiative recombination), *B*_CL_ (radiative recombination), and *C*_CL_ (Auger recombination). See also Figure S7. (h) Analysis of the spontaneous emission quantum efficiency (*η*) for edge and core regions of the two segments of the Ge_1‐x_Sn_x_S nanowire shown in (a,d).

STEM‐CL was measured on the nanowire shown in Figure 7, where a non‐dislocated tip and a dislocated segment, shown in Figure 7a,d, respectively, are part of the same wire (see also Figure 2). EDS of Ge_1‐x_Sn_x_S wires carrying homojunctions between dislocated and defect‐free material demonstrates that the non‐dislocated tips have core‐shell structure and composition identical or very close to that of the adjacent dislocated segments. Quantification of the EDS maps in the area of the non‐dislocated tip (Figure S2a) and dislocated segment (Figure S2b) of the same nanowire yields an overall Sn content of 3.4 at. % at the tip and 3.9 at. % in the dislocated part. Thus, the nanowire segment in Figure 7a has overall composition Ge_0.93_Sn_0.07_S, close to the composition of the dislocated segment shown in Figure 7d (Ge_0.92_Sn_0.08_S).

Electron beam current‐dependent CL was measured in both the defect‐free segment and the dislocated segment, in each case integrating spectra with excitation of the edge region (Figure 7b,e) and central region (Figure 5c,f), respectively, to detect possible differences in quantum efficiency between edge and center. The spectra obtained with the same beam current consistently show the same major features, albeit with small differences in characteristics such as the peak intensity. Present in all spectra, for instance, is a major peak at ∼1.56 eV photon energy, coinciding with the expected band‐edge emission of Ge_1‐x_Sn_x_S alloys (see Figures 5 and 6). All spectra also have a characteristic shoulder toward higher photon energy, as seen above (Figures 5, 6), which likely results from direct transitions between higher‐lying states that are separated by energies greater than the fundamental bandgap. The fact that the high‐energy shoulder is generally present, notably also in the defect‐free parts of our nanowires (see Figure 7), largely rules out other possible explanations, such as bandgap changes due to composition variations or long‐range strain fields near the dislocation.

The electron beam current‐dependent CL spectra were analyzed by determining the integrated light emission (i.e., the area under each spectrum), and fitting plots of beam current versus integrated CL signal with three components corresponding to nonradiative (Shockley‐Read‐Hall, SRH), radiative, and Auger recombination, represented by parameters *A*_CL_ (SRH), *B*_CL_ (radiative) and *C*_CL_ (Auger), respectively (see Figure S7). The exciting electron beam current (*I*_CL_) is expressed in terms of different powers of the CL signal (*L*_CL_) using these parameters: $I_{CL}=A_{CL}{\left(L_{CL}\right)}^{1/2}+B_{CL}L_{CL}+C_{CL}{\left(L_{CL}\right)}^{3/2}$ (Figure 7g and Figure S7). From the *A*_CL_, *B*_CL_, and *C*_CL_ coefficients obtained by least‐squares fitting, the spontaneous emission quantum efficiency, η, can then be determined via $\eta ={\left[1+\frac{A_{CL}}{B_{CL}}\left(1-\gamma_{r}\right){\left(L_{CL}\right)}^{-1/2}+\frac{C_{CL}}{B_{CL}}\left(1-\gamma_{r}\right){\left(L_{CL}\right)}^{1/2}\right]}^{-1}$ (Figure 7h) [34]. In the present study, the term γ_r_, representing the fraction of the spontaneous emission reabsorbed by the active region, was set to zero so that the obtained η represents a lower limit to the actual quantum efficiency.

Performing this analysis on both non‐dislocated and dislocated parts of the same nanowire, the following results are obtained. The non‐dislocated Ge_1‐x_Sn_x_S core‐shell nanowire segments show a high room‐temperature quantum efficiency, *η* ≈ 0.98 and *η* ≈ 0.90, for excitation at the edge and center, respectively. Importantly, both edge and central spectra show minimal SRH nonradiative recombination (i.e., small *A*_CL_; see Figure S7a,b)). The minor reduction in quantum efficiency at the center can be explained by a slight increase in Auger recombination (i.e., larger *C*_CL_), likely due to differences in Auger lifetime between the Ge_1‐x_Sn_x_S alloys that make up the nanowire core and shell.

A distinctly different behavior is found in the nanowire segment that hosts a single helical dislocation. Here, electron beam current‐dependent CL measurements yield a substantially reduced spontaneous emission quantum efficiency, *η* ≈ 0.55 and *η* ≈ 0.20, for excitation in the edge region and center, respectively. The recombination parameters obtained from the fits in Figure S7c,d, help explain this sharp drop in radiative recombination arising with the presence of the helical dislocation. For excitation at the edge, a distinct increase in nonradiative (SRH) recombination (represented by the parameter *A*_CL_) is observed, compared to the measurements on the defect‐free segment (Figure S7c). Measurements in the central region, that is, in close vicinity of the helical dislocation, show even more efficient nonradiative recombination (i.e., larger *A*_CL_; see Figure S7d), which translates into a further reduction in the spontaneous emission quantum efficiency, η.

Adding to prior work that found negligible effect of pure screw dislocations on the spontaneous emission quantum efficiency, that is, found screw dislocations to be benign without introducing deep levels promoting nonradiative recombination [29, 38], the present beam current‐dependent CL measurements comparing defect‐free and dislocated segments clearly demonstrate an increase in nonradiative recombination due to the introduction of an edge‐dislocation component, that is, the conversion of an incipient screw into a helical dislocation. The observed decrease in spontaneous emission quantum efficiency indicates that the edge component of the mixed helical dislocation opens a nonradiative recombination channel that competes with radiative recombination for band‐edge electron‐hole pairs. The observed behavior is therefore consistent with the introduction of gap states by the edge component of the helical dislocation, analogous to 3D semiconductors [39, 40]. It needs to be emphasized that the electron beam current dependent CL analysis presented here goes beyond measuring (or mapping) the intensity of the emitted light [24, 25, 26, 27, 28, 42], thus providing a quantitative local probe of the competition between radiative recombination and different (SRH, Auger) nonradiative recombination mechanisms.

Remarkably, the local STEM‐CL measurements with excitation by a nanometer‐scale electron beam even detect the difference between excitation close to and further away from the dislocation, with the lowest quantum efficiency (i.e., fastest SRH nonradiative recombination) found for electron‐hole pairs excited in the immediate vicinity of the dislocation core. The surprisingly large difference in quantum efficiency between center and periphery can further be explained by the expected bandgap narrowing accompanying the increase in Sn content, *x*, of the Ge_1‐x_Sn_x_S alloys in the shell. The resulting potential barrier impedes the flow of electron‐hole pairs excited at the edge towards the dislocation line, thereby reducing their nonradiative recombination at the dislocation line.

## 3 Conclusions

We demonstrated the controlled formation of Ge_1‐x_Sn_x_S alloy core‐shell nanowires containing individual mixed (helical) dislocations and used cathodoluminescence spectroscopy excited by the nanometer‐focused electron beam in STEM to analyze their optoelectronic properties. The nanowires were prepared in a single‐step VLS growth process using GeS and SnS precursors, where a core‐shell structure is formed by spontaneous phase separation between the poorly miscible GeS and SnS. The amount of Sn incorporated, controlled by the relative vapor pressure of the precursors during growth, provides a tunable axial core‐shell lattice mismatch that is relieved by introduction of edge dislocations. The edge dislocations, in turn, transform the pure axial screw dislocations present in pure GeS nanowires into helical dislocations with tunable geometry (radius, pitch), and hence selectable ratio between screw‐ and edge Burgers vector components. With overall diameters of ∼100 nm, the nanowires provide a small‐volume host matrix where emerging dislocation‐induced phenomena can dominate the functional properties. Together with the ability of creating homojunctions connecting dislocated and defect‐free segments in the same core‐shell nanowires, this provided unique opportunities for investigating the optoelectronic effects of single helical dislocations.

Here, the effects of individual dislocations were probed by STEM‐CL measurements, in which spectra obtained at different positions across single wires demonstrated changes in spectral line shape and substantial emission quenching in the immediate vicinity of the dislocation line. Electron beam current dependent CL spectra were measured and analyzed to determine the spontaneous emission quantum efficiency, again locally as a function of position within individual nanowires as well as comparing defect‐free and dislocated sections of the same nanowire. The measurements show predominant radiative recombination with near‐unity quantum efficiency in the defect‐free part of the nanowires. In the presence of a single mixed dislocation, nonradiative recombination is promoted by the presence of the line defect with finite edge Burgers vector component. For electron‐hole pairs excited in the shell, that is, farthest from the dislocation line, there is still a substantial fraction of radiative recombination as shown by a quantum efficiency above 50%. In the central region, that is, immediately adjacent to the dislocation line, however, radiative recombination is largely quenched and the quantum efficiency drops to 20%. These findings are consistent with the introduction of defect induced deep‐level gap states, correlated with the appearance of an edge component in the helical dislocations. Generally, such gap states originate from dangling bonds, strain, or composition fluctuations near the dislocation core. For van der Waals crystals, dislocation geometries without equivalent in 3D crystals are still emerging [43, 44], and questions such as the core structure [43, 45, 46] as well as the role of dislocations in plasticity [47, 48] are topics of active investigation. Within the context of functional properties of single dislocations, identifying the structure and electronic effects of mixed dislocations with different screw/edge ratios in layered Ge_1‐x_Sn_x_S are clearly important goals for future research.

Overall, the reported results represent a major advance in our ability to create nanostructures containing single dislocations with tunable Burgers vector and probing the effect of the dislocation on optoelectronics, paving the way for the use of dislocations as future elements of technology.

## 4 Materials and Methods

Ge_1‐x_Sn_x_S nanowires were synthesized by vapor‐liquid‐solid growth involving simultaneous vapor transport from two powder precursors, GeS (99.99%, ALB) and SnS (99.99%, ALB), in a pumped two‐zone quartz‐tube reactor. The substrates were Si(100) wafers covered by magnetron sputtering with thin (2‐4 nm) Au films, which dewetted at the growth temperature. The first (upstream) zone of the reactor containing SnS powder (150 mg) in a quartz boat was heated to 650°C–670°C while a second quartz boat with GeS powder (250 mg), located outside the first zone, was heated to ∼430°C–450°C. These conditions yield vapor pressures of ∼250 mTorr for GeS (at 450°C) and ∼100 mTorr for SnS (at 670°C) [49]. The substrate was held in the second (downstream) temperature‐controlled zone heated to 275°C. During growth, the quartz reactor was pumped by a mechanical pump and a carrier gas (Ar; 99.9999%, Matheson) flow was maintained at 60 standard cubic centimeters per minute and a pressure of 20 mTorr. The growth resulted in the formation of forests of core‐shell nanowires with typical lengths of several tens of micrometers.

Structure and morphology of the nanowires were investigated by (scanning) transmission electron microscopy ((S)TEM) imaging and nanobeam electron diffraction in an FEI Talos F200X microscope at 200 kV. For all (S)TEM experiments, the as‐grown nanowires were dry‐transferred to lacey carbon support grids. The composition and distribution of different elements in the nanowires were determined from STEM‐EDS maps and spectra. Cathodoluminescence spectroscopy was performed in STEM mode (STEM‐CL) using a Gatan Vulcan CL holder at room temperature, 200 keV electron energy, and variable incident electron beam currents ranging from 247 to 1070 pA. Panchromatic CL maps were obtained by measuring broadband (400–1000 nm wavelength) light emission across a 512 × 512 pixel image with an integration time of 1.28 ms per pixel. Hyperspectral linescans were acquired by displacing the focused electron beam in predefined equal steps across single nanowires and acquiring full CL spectra at each beam position (integration time: 10 s per spectrum). CL spectra were obtained using a 300 mm Czerny‐Turner spectrometer and a thermoelectric‐cooled back‐illuminated Si CCD detector (1340 × 100 array). The spontaneous emission quantum efficiency was determined by adding 15 individual spectra excited in the core and shell regions, respectively, as shown in Figure 7 and discussed in the associated manuscript text.

## Conflicts of Interest

The authors declare no conflicts of interest.

## Supporting information




**Supporting File**: smll74258‐sup‐0001‐SuppMat.docx.

## Data Availability

The data supporting this article has been included as part of the Supporting Information.

## References

[1] F. C. Frank , “The Influence of Dislocations on Crystal Growth,” Discussions of the Faraday Society 5 (1949): 48–54, 10.1039/df9490500048.

[2] L. Hu , H. Huang , Z. Wang , et al., “Ubiquitous Spin‐Orbit Coupling in a Screw Dislocation With High Spin Coherency,” Physical Review Letters 121 (2018): 066401, 10.1103/PhysRevLett.121.066401. 30141639

[3] S. Rex and R. Willa , “A Topological Flux Trap: Majorana Bound States at Screw Dislocations,” New Journal of Physics 24 (2022): 053057, 10.1088/1367-2630/ac61cf.

[4] M. N. Chernodub and M. A. Zubkov , “Chiral Anomaly in Dirac Semimetals due to Dislocations,” Physical Review B 95 (2017): 115410, 10.1103/PhysRevB.95.115410.

[5] H. Sumiyoshi and S. Fujimoto , “Torsional Chiral Magnetic Effect in a Weyl Semimetal With a Topological Defect,” Physical Review Letters 116 (2016): 166601, 10.1103/PhysRevLett.116.166601. 27152814

[6] R. Soto‐Garrido , E. Muñoz , and V. Juričić , “Dislocation Defect as a Bulk Probe of Monopole Charge of Multi‐Weyl Semimetals,” Physical Review Research 2 (2020): 012043, 10.1103/PhysRevResearch.2.012043.

[7] Y. Ran , Y. Zhang , and A. Vishwanath , “One‐dimensional Topologically Protected Modes in Topological Insulators With Lattice Dislocations,” Nature Physics 5 (2009): 298–303, 10.1038/nphys1220.

[8] M. Li , Y. Tsurimaki , Q. Meng , et al., “Theory of Electron–phonon–dislon Interacting System—Toward a Quantized Theory of Dislocations,” New Journal of Physics 20 (2018): 023010, 10.1088/1367-2630/aaa383.

[9] M. Li , “Quantized Dislocations,” Journal of Physics: Condensed Matter 31 (2019): 083001. 30524003 10.1088/1361-648X/aaf6e1

[10] C.‐L. Fu and M. Li , “Oscillatory Deviations From Matthiessen's Rule due to Interacting Dislocations,” Journal of Physics: Condensed Matter 29 (2017): 325702. 28708604 10.1088/1361-648X/aa7955

[11] S. Hu , H. Zhang , S. Xiong , et al., “Screw Dislocation Induced Phonon Transport Suppression in SiGe Superlattices,” Physical Review B 100 (2019): 075432, 10.1103/PhysRevB.100.075432.

[12] N. Y. Fogel , A. S. Pokhila , Y. V. Bomze , A. Y. Sipatov , A. I. Fedorenko , and R. I. Shekhter , “Novel Superconducting Semiconducting Superlattices: Dislocation‐Induced Superconductivity?,” Physical Review Letters 86 (2001): 512–515, 10.1103/PhysRevLett.86.512. 11177868

[13] R. Ai , X. Cui , Y. Li , and X. Zhuo , “Local Strain Engineering of Two‐Dimensional Transition Metal Dichalcogenides towards Quantum Emitters,” Nano‐Micro Letters 17 (2025): 104, 10.1007/s40820-024-01611-1. 39777585 PMC11711739

[14] J. M. Kim , M. F. Haque , E. Y. Hsieh , et al., “Strain Engineering of Low‐Dimensional Materials for Emerging Quantum Phenomena and Functionalities,” Advanced Materials 35 (2023): 2107362, 10.1002/adma.202107362. 34866241

[15] X. Zhou , Z. Zhang , and W. Guo , “Dislocations as Single Photon Sources in Two‐Dimensional Semiconductors,” Nano Letters 20 (2020): 4136–4143, 10.1021/acs.nanolett.9b05305. 32453959

[16] G. Zhang , Y. Cheng , J.‐P. Chou , and A. Gali , “Material Platforms for Defect Qubits and Single‐photon Emitters,” Applied Physics Reviews 7 (2020): 031308, 10.1063/5.0006075.

[17] A. Chatterjee , A. Biswas , A. S. Fuhr , et al., “Room‐temperature High‐purity Single‐photon Emission From Carbon‐doped Boron Nitride Thin Films,” Science Advances 11 (2025): adv2899, 10.1126/sciadv.adv2899. PMC13108819 40540575

[18] M. Reiche , M. Kittler , W. Erfurth , et al., “On the Electronic Properties of a Single Dislocation,” Journal of Applied Physics 115 (2014): 194303, 10.1063/1.4876265.

[19] M. Soleimany , T. Frömling , J. Rödel , and M. Alexe , “Dislocation‐Induced Local and Global Photoconductivity Enhancement and Mechanisms in Iron‐Doped SrTiO_3_ ,” Advanced Functional Materials 35 (2025): 2417952, 10.1002/adfm.202417952.

[20] M. Kissel , L. Porz , T. Frömling , A. Nakamura , J. Rödel , and M. Alexe , “Enhanced Photoconductivity at Dislocations in SrTiO_3_ ,” Advanced Materials 34 (2022): 2203032, 10.1002/adma.202203032. 35727056

[21] M. Kittler and M. Reiche , “Dislocations as Active Components in Novel Silicon Devices,” Advanced Engineering Materials 11 (2009): 249–258, 10.1002/adem.200800283.

[22] M. Kittler , X. Yu , T. Mchedlidze , et al., “Regular Dislocation Networks in Silicon as a Tool for Nanostructure Devices Used in Optics,” Biology, and Electronics Small 3 (2007): 964–973. 17429814 10.1002/smll.200600539

[23] F. Zhuo , X. Zhou , F. Dietrich , et al., “Dislocation Density‐Mediated Functionality in Single‐Crystal BaTiO_3_ ,” Advanced Science 11 (2024): 2403550. 38885353 10.1002/advs.202403550 PMC11336959

[24] P. M. Petroff , R. A. Logan , and A. Savage , “Nonradiative Recombination at Dislocations in III‐V Compound Semiconductors,” Physical Review Letters 44 (1980): 287–291, 10.1103/PhysRevLett.44.287.

[25] S. J. Pennycook , L. M. Brown , and A. J. Craven , “Observation of Cathodoluminescence at Single Dislocations by STEM,” Philosophical Magazine A 41 (1980): 589–600, 10.1080/01418618008239335.

[26] S. Myhajlenko , J. L. Batstone , H. J. Hutchinson , and J. W. Steeds , “Luminescence Studies of Individual Dislocations in II‐VI (ZnSe) and III‐V (InP) Semiconductors,” Journal of Physics C: Solid State Physics 17 (1984): 6477–6492, 10.1088/0022-3719/17/35/017.

[27] V. M. Kaganer , J. Lähnemann , C. Pfüller , K. K. Sabelfeld , A. E. Kireeva , and O. Brandt , “Determination of the Carrier Diffusion Length in GaN From Cathodoluminescence Maps Around Threading Dislocations: Fallacies and Opportunities,” Physical Review Applied 12 (2019): 054038, 10.1103/PhysRevApplied.12.054038.

[28] J. Lähnemann , V. M. Kaganer , K. K. Sabelfeld , et al., “Carrier Diffusion in GaN: A Cathodoluminescence Study. III. Nature of Nonradiative Recombination at Threading Dislocations,” Physical Review Applied 17 (2022): 024019, 10.1103/PhysRevApplied.17.024019.

[29] P. Sutter , R. R. Unocic , and E. Sutter , “Tuning of Single Mixed (Helical) Dislocations in Core–Shell van der Waals Nanowires,” Journal of the American Chemical Society 145 (2023): 20503–20510, 10.1021/jacs.3c06469. 37695639

[30] P. Sutter , S. Wimer , and E. Sutter , “Chiral Twisted van der Waals Nanowires,” Nature 570 (2019): 354–357, 10.1038/s41586-019-1147-x. 31011183

[31] E. Sutter and P. Sutter , “Ultrathin Twisted Germanium Sulfide van der Waals Nanowires by Bismuth Catalyzed Vapor–Liquid–Solid Growth,” Small 17 (2021): 2104784, 10.1002/smll.202104784. 34655159

[32] P. Sutter and E. Sutter , “Tunable 1D van der Waals Nanostructures by Vapor–Liquid–Solid Growth,” Accounts of Chemical Research 56 (2023): 3235–3245, 10.1021/acs.accounts.3c00502. 37938893

[33] E. Sutter and P. Sutter , “GeS Mixed‐dimensional 1D Nanowire–2D Plate Heterostructures on van der Waals Substrates,” Materials Advances 7 (2026): 255–264, 10.1039/D5MA00694E.

[34] E. Sutter , J. S. French , H.‐P. Komsa , and P. Sutter , “1D Germanium Sulfide van der Waals Bicrystals by Vapor–Liquid–Solid Growth,” ACS Nano 16 (2022): 3735–3743, 10.1021/acsnano.1c07349. 35147417

[35] E. Sutter , J. S. French , and P. Sutter , “Tunable Layer Orientation and Morphology in Vapor–Liquid–Solid Growth of One‐Dimensional GeS van der Waals Nanostructures,” Chemistry of Materials 33 (2021): 3980–3988, 10.1021/acs.chemmater.1c00289.

[36] P. Sutter , J.‐C. Idrobo , and E. Sutter , “Van der Waals Nanowires With Continuously Variable Interlayer Twist and Twist Homojunctions,” Advanced Functional Materials 31 (2021): 2006412, 10.1002/adfm.202006412.

[37] J. D. Eshelby , “Screw Dislocations in Thin Rods,” Journal of Applied Physics 24 (1953): 176–179, 10.1063/1.1721234.

[38] C. V. Collins and M. H. Miles , “Electrical Effects of Screw Dislocations in Germanium,” Journal of Applied Physics 42 (1971): 5644–5648, 10.1063/1.1659994.

[39] M. Jastrzębska and T. Figielski , “Trapping Processes at Dislocations in Plastically Bent Germanium,” Physica Status Solidi 14 (1966): 381–390.

[40] M. H. Miles , “Extrinsic Photoconductivity From Edge Dislocations in Germanium,” Journal of Applied Physics 40 (1969): 2720–2724, 10.1063/1.1658068.

[41] M. A. Reshchikov and H. Morkoç , “Luminescence Properties of Defects in GaN,” Journal of Applied Physics 97 (2005): 061301, 10.1063/1.1868059.

[42] A. Jakubowicz , “Theory of Cathodoluminescence Contrast From Localized Defects in Semiconductors,” Journal of Applied Physics 59 (1986): 2205–2209, 10.1063/1.336359.

[43] A. Kushima , X. Qian , P. Zhao , S. Zhang , and J. Li , “Ripplocations in van der Waals Layers,” Nano Letters 15 (2015): 1302–1308, 10.1021/nl5045082. 25562795

[44] T. H. Yang , B.‐W. Liang , H.‐C. Hu , et al., “Ferroelectric Transistors Based on Shear‐transformation‐mediated Rhombohedral‐stacked Molybdenum Disulfide,” Nature Electronics 7 (2024): 29–38, 10.1038/s41928-023-01073-0.

[45] D. L. Medlin , N. Yang , C. D. Spataru , L. M. Hale , and Y. Mishin , “Unraveling the Dislocation Core Structure at a van der Waals Gap in Bismuth Telluride,” Nature Communications 10 (2019): 1820, 10.1038/s41467-019-09815-5. PMC6478677 31015459

[46] T. H. Ly , J. Zhao , D. H. Keum , Q. Deng , Z. Yu , and Y. H. Lee , “Hyperdislocations in van der Waals Layered Materials,” Nano Letters 16 (2016): 7807–7813, 10.1021/acs.nanolett.6b04002. 27960496

[47] T.‐R. Wei , M. Jin , Y. Wang , et al., “Exceptional Plasticity in the Bulk Single‐crystalline van der Waals Semiconductor InSe,” Science 369 (2020): 542–545, 10.1126/science.aba9778. 32732421

[48] A. N. Rudenko , M. I. Katsnelson , and Y. N. Gornostyrev , “Dislocation Structure and Mobility in the Layered Semiconductor InSe: A First‐principles Study,” 2D Materials 8 (2021): 045028, 10.1088/2053-1583/ac207b.

[49] K. C. Mills , Thermodynamic Data for Inorganic Sulphides, Selenides and Tellurides (Butterworths, 1974), 328–332.

