# Supplementary material for: Optoelectronics of Single Mixed Dislocations in Van Der Waals Core‐Shell Nanowires
Source: Small. 2026 Jun 19;22(44):e74258. doi: 10.1002/smll.74258 (PMC13450293; doi:10.1002/smll.74258)
Supplement: Supplementary file 1 [file SMLL-22-e74258-s001.docx]

Supplementary Information

**Optoelectronics of Single Mixed Dislocations in van der Waals Core-Shell Nanowires**

Eli Sutter,^1,*^ Peter Sutter^2^

^1^Department of Mechanical & Materials Engineering, University of Nebraska-Lincoln, Lincoln, Nebraska 68588, United States

^2^Department of Electrical & Computer Engineering, University of Nebraska-Lincoln, Lincoln, Nebraska 68588, United States

**1. Supplementary Figures**

**Figure S1. Core-shell van der Waals nanowires. (a)** Schematic of a defect-free layered core-shell nanowire. **(b)** Layered core-shell nanowire carrying a single helical dislocation (core: red; shell: green). Helix geometry: radius *r*_0_; pitch λ; angle *θ* between the Burgers vector and the helical dislocation line.

**Figure S2. Composition of the segments of nanowires without dislocations and with helical dislocations. (a)** HAADF-STEM image and **(b)** **– (d).** EDS elemental maps of the distribution of germanium, tin and sulfur in the segment without dislocation at the tip of a typical nanowire. **(e)** HAADF-STEM image along with germanium, tin and sulfur EDS elemental maps of the segment of the nanowire containing a helical dislocation.

**Figure S3. Composition of a thin alloy core-shell nanowire containing helical dislocations. (a)** HAADF-STEM image and **(b)** **– (d).** EDS elemental and combined maps of the distribution of germanium, tin and sulfur in the nanowire with overall composition Ge_0.72_Sn_0.28_S.

**Figure S4. Estimate of the core composition from core and shell EDS spectra and the core/shell dimensions.** Left: EDS measurement with exciting electron beam positioned in the shell. Center: EDS measurement with exciting electron beam traversing core and shell.

- Sn content of the shell: *x*_Sn_^shell^ obtained directly from EDS spectrum (core only).
- Estimated Sn content of the core: *x*_Sn_ = [*x*_Sn_^core+shell^ (2 *d*_shell_ + *d*_core_) - 2*d*_shell_ *x*_Sn_^shell^]/*d*_core_

**Figure S5. Reproducibility of the spectral lineshape along a nanowire. (a)** STEM image of part of a Ge_1-x_Sn_x_S nanowire carrying a single helical dislocation. **(b)** Hyperspectral CL linescan along the arrow in (a). Top: Example of a single spectrum.

**Figure S6. Differences in emitted light intensity close to and far from the helical dislocation line.** **(a)** STEM image of a nanowire carrying a single helical dislocation. **(b)** Hyperspectral CL linescan obtained along the arrow shown in panel (a). **(c)** Intensity profile of the main peak centered at ~1.6 eV photon energy.

**Figure S7. Fits of electron beam current vs. integrated CL signal. (a)** Current-dependent CL data obtained from the shell of the defect-free nanowire segment (Fig. 7 (b)). **(b)** Current-dependent CL data obtained in the center of the defect-free nanowire segment (Fig. 7 (c)). **(c)** Current-dependent CL data obtained from the shell of the dislocated nanowire segment (Fig. 7 (e)). **(d)** Current-dependent CL data obtained in the center of the dislocated nanowire segment (Fig. 7 (f)).

Fits in the above plots are to the relationship $I_{\mathrm{CL}}=A_{\mathrm{CL}}\left( L_{\mathrm{CL}} \right)^{\frac{1}{2}}+B_{\mathrm{CL}}L_{\mathrm{CL}}+C_{\mathrm{CL}}\left( L_{\mathrm{CL}} \right)^{\frac{3}{2}}$, where *I*_CL_ denotes the exciting beam current and *L*_CL_ is the integrated CL signal. Parameters *A*_CL_, *B*_CL_, and *C*_CL_ represent nonradiative (Shockley-Read-Hall, SRH) recombination, radiative recombination, and Auger recombination, respectively. From the (*A*_CL_, *B*_CL_, *C*_CL_) parameters, the spontaneous emission quantum efficiency (*η*) is determined as:

$\eta=\left[ 1+\frac{A_{CL}}{B_{CL}}\left( 1-\gamma_{r} \right)\left( L_{CL} \right)^{-\frac{1}{2}}+\frac{C_{CL}}{B_{CL}}\left( 1-\gamma_{r} \right)\left( L_{CL} \right)^{\frac{1}{2}} \right]^{-1}$,

where *γ*_r_ denotes the fraction of the spontaneous emission that is reabsorbed by the active region. The results of this analysis are shown in Fig. 7 (g) for the most conservative choice of the photon recycling factor, *γ*_r_ = 0. Note that the plots shown in panels (a) – (d) generally have very low Auger recombination parameters (*C*_CL_). The competition between SRH nonradiative (*A*_CL_) and radiative recombination (*B*_CL_) is captured in the fact that *I*_CL_ ~ *L*_CL_ for radiative and *I*_CL_ ~ √ *L*_CL_ for SRH recombination. Although the difference may appear subtle at first glance, the fits reveal clear differences with predominant radiative recombination in panels (a) and (b) and majority SRH nonradiative recombination in (c) and (d).

**2. Data**

**2.1. CL spectrum excited at the nanowire edge (Fig. 5 (c), top)**

**Energy (eV)	Intensity (counts)**

1.1973	0.22692

1.2057	0.29999

1.2141	0.35706

1.2225	0.58942

1.2309	0.69843

1.2393	0.78408

1.2477	1.0936

1.2561	1.2094

1.2645	1.1889

1.2729	1.5651

1.2813	1.6018

1.2896	1.9332

1.2980	2.1872

1.3064	2.4313

1.3148	2.8404

1.3232	3.0257

1.3316	3.4365

1.3400	3.6431

1.3484	3.7755

1.3568	4.0134

1.3652	4.1030

1.3736	4.2799

1.3820	4.6074

1.3903	4.7773

1.3987	5.1571

1.4071	5.2066

1.4155	5.1516

1.4239	5.6367

1.4323	5.6978

1.4407	5.8671

1.4491	5.7488

1.4575	6.1329

1.4659	6.1694

1.4743	6.2367

1.4827	6.8517

1.4911	6.8711

1.4995	6.8126

1.5078	7.1852

1.5162	7.2060

1.5246	7.5231

1.5330	7.4576

1.5414	7.6378

1.5498	7.7206

1.5582	7.9115

1.5666	7.8388

1.5750	7.8386

1.5834	7.6893

1.5918	7.4798

1.6002	7.5121

1.6086	7.3046

1.6169	7.0045

1.6253	6.7136

1.6337	6.5031

1.6421	6.6352

1.6505	6.3311

1.6589	6.1040

1.6673	5.8715

1.6757	5.7482

1.6841	5.5904

1.6925	5.4737

1.7009	5.3186

1.7093	5.4483

1.7177	5.1182

1.7260	5.3035

1.7344	5.0439

1.7428	5.2161

1.7512	5.0893

1.7596	5.1553

1.7680	5.1770

1.7764	5.2494

1.7848	5.1442

1.7932	5.3221

1.8016	5.2480

1.8100	5.2813

1.8184	5.2621

1.8268	5.2159

1.8351	5.1907

1.8435	5.0183

1.8519	4.9289

1.8603	4.8887

1.8687	4.7890

1.8771	4.6802

1.8855	4.6313

1.8939	4.4858

1.9023	4.3026

1.9107	4.1008

1.9191	3.9070

1.9275	3.7577

1.9359	3.6608

1.9442	3.5790

1.9526	3.3526

1.9610	3.2552

1.9694	3.1168

1.9778	2.9658

1.9862	2.8904

1.9946	2.8166

2.0030	2.7288

2.0114	2.6538

2.0198	2.5820

2.0282	2.4525

2.0366	2.3831

2.0450	2.3783

2.0533	2.4136

2.0617	2.4172

2.0701	2.3328

2.0785	2.2984

2.0869	2.3408

2.0953	2.2391

2.1037	2.2118

2.1121	2.1991

2.1205	2.0892

2.1289	2.0702

2.1373	2.0669

2.1457	2.0398

2.1541	1.9753

2.1624	1.9359

2.1708	1.9568

2.1792	1.9496

2.1876	1.9257

2.1960	1.8768

2.2044	1.8289

2.2128	1.7978

2.2212	1.8329

2.2296	1.8155

2.2380	1.7663

2.2464	1.8118

2.2548	1.7913

2.2632	1.6693

2.2715	1.6430

2.2799	1.6583

2.2883	1.7012

2.2967	1.6885

2.3051	1.7231

2.3135	1.6708

2.3219	1.6638

2.3303	1.6430

2.3387	1.6874

2.3471	1.7015

2.3555	1.6663

2.3639	1.6987

2.3723	1.6442

2.3807	1.5967

2.3890	1.5595

2.3974	1.5689

2.4058	1.5905

2.4142	1.6045

2.4226	1.5827

2.4310	1.5636

2.4394	1.5510

2.4478	1.5421

2.4562	1.5390

2.4646	1.5430

2.4730	1.5557

2.4814	1.5452

2.4897	1.5103

2.4981	1.4915

2.5065	1.4887

2.5149	1.4878

2.5233	1.4887

2.5317	1.4810

2.5401	1.4626

2.5485	1.4444

2.5569	1.4263

2.5653	1.4114

2.5737	1.4042

2.5821	1.3983

2.5905	1.3993

**2.2. CL spectrum excited at the nanowire center (Fig. 5 (c), bottom)**

**Energy (eV)	Intensity (counts)**

1.1973	0.22076

1.2057	0.20900

1.2141	0.32889

1.2225	0.53376

1.2309	0.70619

1.2393	0.74271

1.2477	1.0056

1.2561	1.0833

1.2645	1.1711

1.2729	1.4288

1.2813	1.5912

1.2896	1.8343

1.2980	2.1675

1.3064	2.3412

1.3148	2.4861

1.3232	2.7742

1.3316	3.1623

1.3400	3.3276

1.3484	3.5496

1.3568	3.5849

1.3652	3.6989

1.3736	3.7841

1.3820	3.8733

1.3903	3.9356

1.3987	3.9352

1.4071	4.0210

1.4155	4.0885

1.4239	4.0843

1.4323	4.0615

1.4407	4.0151

1.4491	4.0699

1.4575	4.0287

1.4659	3.9915

1.4743	3.9547

1.4827	4.2588

1.4911	4.1238

1.4995	4.1616

1.5078	4.2115

1.5162	4.3029

1.5246	4.3022

1.5330	4.4527

1.5414	4.4528

1.5498	4.6420

1.5582	4.4980

1.5666	4.7856

1.5750	4.7772

1.5834	4.7720

1.5918	4.9761

1.6002	4.9030

1.6086	4.8359

1.6169	4.9133

1.6253	4.9901

1.6337	5.0572

1.6421	4.9994

1.6505	5.0396

1.6589	5.0289

1.6673	4.8693

1.6757	4.8551

1.6841	4.8465

1.6925	4.7042

1.7009	4.6555

1.7093	4.6731

1.7177	4.5258

1.7260	4.4529

1.7344	4.3731

1.7428	4.3678

1.7512	4.1989

1.7596	4.3040

1.7680	4.1382

1.7764	4.0213

1.7848	4.0325

1.7932	3.8550

1.8016	3.8092

1.8100	3.7472

1.8184	3.6755

1.8268	3.5841

1.8351	3.5333

1.8435	3.4130

1.8519	3.3304

1.8603	3.2871

1.8687	3.2158

1.8771	3.1373

1.8855	3.1259

1.8939	3.0027

1.9023	2.8369

1.9107	2.7384

1.9191	2.6039

1.9275	2.5094

1.9359	2.4756

1.9442	2.3762

1.9526	2.1785

1.9610	2.1474

1.9694	2.0561

1.9778	1.9664

1.9862	2.0083

1.9946	1.8720

2.0030	1.7743

2.0114	1.7787

2.0198	1.8278

2.0282	1.6781

2.0366	1.6443

2.0450	1.6219

2.0533	1.6037

2.0617	1.6256

2.0701	1.6365

2.0785	1.6607

2.0869	1.6959

2.0953	1.5666

2.1037	1.5197

2.1121	1.5116

2.1205	1.4786

2.1289	1.4887

2.1373	1.4896

2.1457	1.4432

2.1541	1.4711

2.1624	1.5144

2.1708	1.5600

2.1792	1.5572

2.1876	1.5272

2.1960	1.4576

2.2044	1.4394

2.2128	1.4385

2.2212	1.4417

2.2296	1.4688

2.2380	1.5102

2.2464	1.5158

2.2548	1.4921

2.2632	1.4233

2.2715	1.4165

2.2799	1.4313

2.2883	1.4563

2.2967	1.4732

2.3051	1.4787

2.3135	1.4740

2.3219	1.4650

2.3303	1.4549

2.3387	1.4480

2.3471	1.4514

2.3555	1.4323

2.3639	1.3880

2.3723	1.3769

2.3807	1.3815

2.3890	1.3673

2.3974	1.3511

2.4058	1.3612

2.4142	1.3724

2.4226	1.3907

2.4310	1.3986

2.4394	1.3816

2.4478	1.3751

2.4562	1.3855

2.4646	1.3779

2.4730	1.3480

2.4814	1.3162

2.4897	1.2823

2.4981	1.2933

2.5065	1.3488

2.5149	1.3585

2.5233	1.3207

2.5317	1.3000

2.5401	1.3001

2.5485	1.2964

2.5569	1.2866

2.5653	1.2656

2.5737	1.2164

2.5821	1.1815

2.5905	1.2201

**2.3. Beam current-dependent CL spectra, non-dislocated, edge (Fig. 7 (b))**

| **Photon energy (eV)** | **247 pA** | **302 pA** | **380 pA** | **452 pA** | **668 pA** | **1070 pA** |
| --- | --- | --- | --- | --- | --- | --- |
| 1.1973 | 589.01 | 490.49 | 270.43 | 554.47 | 276.51 | 4.0897 |
| 1.2057 | 513.79 | 265.06 | 287.25 | 564.42 | 636.49 | 986.66 |
| 1.2141 | 244.03 | 250.84 | 145.06 | 417.49 | 580.98 | 839.7 |
| 1.2225 | 524.1 | 429.34 | 537.54 | 655.92 | 732.38 | 1344.4 |
| 1.2309 | 110.14 | 96.439 | 166.62 | 364.4 | 556.57 | 1107.9 |
| 1.2393 | 220 | 82.344 | 382.07 | 373.19 | 781.09 | 1660.4 |
| 1.2477 | 1218.6 | 1162.9 | 1162.5 | 1433.8 | 1869.9 | 2571.1 |
| 1.2561 | 360.18 | 438.27 | 682.87 | 678.84 | 1532.8 | 2335.8 |
| 1.2645 | 791.65 | 986.99 | 1237.4 | 1430.5 | 1951.7 | 3029.2 |
| 1.2729 | 542.86 | 805.43 | 778.06 | 942.09 | 1892.7 | 2991.1 |
| 1.2813 | 918.01 | 1065.2 | 1200.2 | 1496.5 | 2505.3 | 3700.4 |
| 1.2896 | 967.1 | 1319.1 | 1251.3 | 1482.6 | 2625.7 | 3885.3 |
| 1.298 | 950.56 | 1073.7 | 1220.7 | 1308.6 | 2925 | 4286.5 |
| 1.3064 | 1190.9 | 1641.3 | 1562.4 | 2010.9 | 3484.9 | 4900.9 |
| 1.3148 | 940.32 | 1306.3 | 1495.4 | 1862.7 | 3410.1 | 4933.3 |
| 1.3232 | 1462.1 | 1710.2 | 2186.6 | 2453.6 | 4423.3 | 5903.1 |
| 1.3316 | 1247.4 | 1334.3 | 1796.2 | 2446.1 | 4207.7 | 5652.4 |
| 1.34 | 2353.7 | 2585.4 | 3035.3 | 3586.5 | 5532.3 | 7214.1 |
| 1.3484 | 1984.4 | 2371 | 2740.4 | 3443.8 | 5303 | 7023.7 |
| 1.3568 | 1875.4 | 2253.7 | 2749.3 | 3258.2 | 5333.7 | 7372 |
| 1.3652 | 2095.8 | 2435.5 | 3183.8 | 4055.8 | 6000.5 | 8259.8 |
| 1.3736 | 2557.8 | 2773 | 3594.7 | 4523.1 | 6542.9 | 8992.1 |
| 1.382 | 2330.7 | 2476.1 | 3308.2 | 4444.1 | 6290.1 | 8988.1 |
| 1.3903 | 2947.4 | 3104 | 4151.8 | 5200.5 | 7116.8 | 10078 |
| 1.3987 | 2825.7 | 2972.5 | 3612 | 4870.8 | 7023.1 | 10304 |
| 1.4071 | 2628 | 3024.5 | 3797.7 | 5095.7 | 7250.6 | 10661 |
| 1.4155 | 2936.1 | 3171.9 | 3855 | 5305.4 | 7393.2 | 11675 |
| 1.4239 | 3271.6 | 3430.6 | 4121.5 | 5473.4 | 7969.4 | 12167 |
| 1.4323 | 2887.1 | 3098.4 | 3955.7 | 5168.3 | 7553.8 | 11905 |
| 1.4407 | 3493.6 | 3706.5 | 4515.8 | 5784 | 8179.2 | 12981 |
| 1.4491 | 3556.6 | 3837.7 | 4439.8 | 5897.3 | 8312.4 | 13110 |
| 1.4575 | 3717.1 | 3790.6 | 4609.3 | 5722.3 | 8155.3 | 13220 |
| 1.4659 | 3470.9 | 3723.9 | 4417.4 | 5958.1 | 8240.8 | 13103 |
| 1.4743 | 3807.2 | 4062.3 | 4792.7 | 6171.4 | 8498.6 | 13387 |
| 1.4827 | 3714.2 | 3949.6 | 4434.4 | 5921.6 | 8169.6 | 13088 |
| 1.4911 | 3818.5 | 4094.8 | 4519.8 | 6155.1 | 8527.9 | 13392 |
| 1.4995 | 3721.7 | 4120.4 | 4584.6 | 6325 | 8732.2 | 13574 |
| 1.5078 | 3987.3 | 4466.6 | 4983.1 | 6491.8 | 9291.3 | 14428 |
| 1.5162 | 4137.3 | 4423.4 | 4756.6 | 6425.3 | 9449.5 | 14693 |
| 1.5246 | 4193.4 | 4413.9 | 4991.4 | 6605.4 | 9783.9 | 15014 |
| 1.533 | 4234.1 | 4823.6 | 5145.4 | 6814.1 | 10158 | 15805 |
| 1.5414 | 4769.5 | 5603.3 | 5711.6 | 7396.8 | 11032 | 16852 |
| 1.5498 | 4484.6 | 5421.3 | 5469.9 | 7164 | 11095 | 17234 |
| 1.5582 | 4554.5 | 5729.6 | 6065.7 | 7688.6 | 11731 | 18161 |
| 1.5666 | 4867.9 | 5857.2 | 6176.7 | 7819.3 | 11985 | 19040 |
| 1.575 | 5023.4 | 5889.4 | 6222.5 | 7836.9 | 12065 | 19347 |
| 1.5834 | 4706.4 | 5656.4 | 6315.6 | 7442.1 | 11746 | 19465 |
| 1.5918 | 4516.6 | 5520.8 | 6020.2 | 7370.4 | 11510 | 19399 |
| 1.6002 | 4137.5 | 5074 | 5809.3 | 6931.3 | 11137 | 19533 |
| 1.6086 | 4066.3 | 5100.2 | 5784 | 6737.3 | 10999 | 19322 |
| 1.6169 | 3833.9 | 4804.1 | 5603 | 6409.4 | 10481 | 18680 |
| 1.6253 | 3999.3 | 5015.3 | 5940.3 | 6398.4 | 10302 | 18373 |
| 1.6337 | 3800.7 | 4453.7 | 6110.4 | 6166 | 9944.1 | 17487 |
| 1.6421 | 3594.2 | 4181.5 | 5926.7 | 5907.3 | 9096.3 | 16864 |
| 1.6505 | 3356.4 | 3891.5 | 5714.8 | 5795.8 | 8773.3 | 15855 |
| 1.6589 | 3222.4 | 3832.2 | 5637.4 | 5554.7 | 8308.4 | 14929 |
| 1.6673 | 2982.9 | 3562.4 | 5530.7 | 5546.4 | 8204.3 | 14317 |
| 1.6757 | 2526.8 | 3126.7 | 4910.1 | 5065.9 | 7579.6 | 13420 |
| 1.6841 | 2577.1 | 3297 | 4873.7 | 5003.8 | 7587.9 | 13333 |
| 1.6925 | 2364.6 | 3027.8 | 4609.1 | 4842.2 | 7272 | 12750 |
| 1.7009 | 2672.1 | 3088.9 | 4774.2 | 4902.8 | 7282.1 | 12564 |
| 1.7093 | 2414.1 | 2800.6 | 4320.9 | 4452.8 | 7188.2 | 12179 |
| 1.7177 | 2656 | 3105.5 | 4554.1 | 4562.4 | 7038.9 | 11796 |
| 1.726 | 2536.3 | 2935.7 | 4139.5 | 4386.3 | 6960.1 | 11644 |
| 1.7344 | 2121.4 | 2442 | 4002.6 | 3777.8 | 6205.4 | 10923 |
| 1.7428 | 2303.2 | 2565.7 | 4113.3 | 4014.4 | 6353.8 | 10708 |
| 1.7512 | 2198.4 | 2489.2 | 3940.6 | 4062.3 | 5973 | 10299 |
| 1.7596 | 2021.4 | 2283.3 | 3681.8 | 3758.7 | 5558.2 | 9554 |
| 1.768 | 2096.9 | 2393.4 | 3575.5 | 3750.5 | 5586.7 | 9441.3 |
| 1.7764 | 2177.6 | 2347.7 | 3732.2 | 3763.3 | 5428.9 | 9182.2 |
| 1.7848 | 2431.9 | 2599.1 | 3895.4 | 4030.5 | 5539.8 | 9072.8 |
| 1.7932 | 2134.8 | 2275.9 | 3439.9 | 3706.9 | 5156.6 | 8779.3 |
| 1.8016 | 2316.6 | 2514.8 | 3369.6 | 3733.3 | 5203.5 | 8678.6 |
| 1.81 | 2076.2 | 2293.3 | 3105.5 | 3563.4 | 4952.5 | 8236.6 |
| 1.8184 | 2161.5 | 2284.6 | 3037.3 | 3666 | 4975.8 | 8291.7 |
| 1.8268 | 2065.1 | 2279.5 | 3033.3 | 3547.5 | 4816.4 | 8211.7 |
| 1.8351 | 1788 | 2102.4 | 2668.7 | 3214.1 | 4738.1 | 8100.1 |
| 1.8435 | 1886 | 2180.5 | 2765.4 | 3220.5 | 4690.6 | 8242.8 |
| 1.8519 | 1922.8 | 1994.3 | 2705.8 | 3110 | 4686.8 | 8172.5 |
| 1.8603 | 1984.4 | 2008.1 | 2724.1 | 3097.2 | 4827.2 | 8270.5 |
| 1.8687 | 2132.5 | 2321.5 | 2905.1 | 3240.1 | 4961.2 | 8568.5 |
| 1.8771 | 2132.9 | 2391.4 | 2861.2 | 3219.1 | 4921.3 | 8692.3 |
| 1.8855 | 1990.7 | 2239.7 | 2701.2 | 3120.5 | 4884.2 | 8840.2 |
| 1.8939 | 1857.8 | 2244.7 | 2546.4 | 3117.7 | 4839.7 | 8677.3 |
| 1.9023 | 1930.5 | 2302.9 | 2732.4 | 3219.1 | 5087.1 | 8815.3 |
| 1.9107 | 2091.5 | 2484.3 | 2875.5 | 3312.1 | 5251 | 8990.5 |
| 1.9191 | 2030 | 2360.7 | 2863 | 3206.7 | 5156.4 | 8983.1 |
| 1.9275 | 2035.8 | 2267 | 2880.9 | 3167.6 | 5163 | 8904.2 |
| 1.9359 | 2201.2 | 2359.6 | 2992.1 | 3272.6 | 5356 | 8820.3 |
| 1.9442 | 2133.7 | 2261.1 | 2995.9 | 3199.6 | 5288.2 | 8613.4 |
| 1.9526 | 2090.6 | 2393.3 | 3065.2 | 3348.5 | 5346.9 | 8595.2 |
| 1.961 | 2027.1 | 2257.4 | 2932.8 | 3268.9 | 5139 | 8509.2 |
| 1.9694 | 2009 | 2196 | 2865.5 | 3222.7 | 5088.1 | 8320.2 |
| 1.9778 | 2011.8 | 2241.6 | 2858.8 | 3224.2 | 5165.7 | 8123.8 |
| 1.9862 | 1985.7 | 2302.5 | 2822.3 | 3195.6 | 5180.8 | 8033.8 |
| 1.9946 | 1969.6 | 2259.8 | 2891.8 | 3096.6 | 5064.6 | 7862.5 |
| 2.003 | 1906.5 | 2250.6 | 2922.3 | 3093.8 | 4948.8 | 7693.6 |
| 2.0114 | 1881.3 | 2218 | 2862.2 | 3076.1 | 4879.5 | 7548.6 |
| 2.0198 | 1897.4 | 2158.3 | 2757.2 | 3028.1 | 4833.6 | 7384.1 |
| 2.0282 | 1874.3 | 2121 | 2802.4 | 3024.5 | 4689.5 | 7038.7 |
| 2.0366 | 1764.5 | 2002.1 | 2760.6 | 2921.3 | 4505.8 | 6920.9 |
| 2.045 | 1738.8 | 1940.3 | 2761 | 2887.5 | 4379.5 | 6864.4 |
| 2.0533 | 1768.4 | 1937.8 | 2778.7 | 2901.4 | 4284.3 | 6807 |
| 2.0617 | 1680.4 | 1937.1 | 2667.7 | 2811.3 | 4091.1 | 6646.2 |
| 2.0701 | 1587.9 | 1803.5 | 2433.9 | 2645.5 | 3857.2 | 6270.9 |
| 2.0785 | 1474.8 | 1668.6 | 2266.4 | 2505 | 3679.8 | 5979.3 |
| 2.0869 | 1343.8 | 1570.5 | 2214.4 | 2418.4 | 3583.9 | 5850.4 |
| 2.0953 | 1338.5 | 1548.8 | 2206.1 | 2392 | 3522.7 | 5807.7 |
| 2.1037 | 1354.2 | 1546.9 | 2160.1 | 2372.8 | 3437 | 5764.7 |
| 2.1121 | 1367.5 | 1546.9 | 2104 | 2346.8 | 3340.5 | 5701.2 |
| 2.1205 | 1329.7 | 1514.6 | 2085.8 | 2282.3 | 3252.7 | 5563.7 |
| 2.1289 | 1083.8 | 1321.7 | 1838.1 | 2038.9 | 2959.3 | 5168.3 |
| 2.1373 | 936.64 | 1200.6 | 1638.2 | 1852.5 | 2746.7 | 4879.8 |
| 2.1457 | 1133.8 | 1335.5 | 1685.7 | 1901.3 | 2836.9 | 4983.7 |
| 2.1541 | 1015.5 | 1219.1 | 1594.3 | 1834.9 | 2692.9 | 4762.3 |
| 2.1624 | 908.85 | 1116.8 | 1517.8 | 1768.2 | 2570.4 | 4570.3 |
| 2.1708 | 938.19 | 1131.7 | 1519 | 1742.3 | 2572.1 | 4550.1 |
| 2.1792 | 892.26 | 1096.3 | 1466.6 | 1694.8 | 2553.4 | 4397.5 |
| 2.1876 | 899.06 | 1080.4 | 1464.1 | 1664.1 | 2560.5 | 4287.5 |
| 2.196 | 1057 | 1130.5 | 1596.6 | 1680.3 | 2634.1 | 4329.7 |
| 2.2044 | 1033.2 | 1120.8 | 1575.1 | 1679 | 2584.5 | 4229.9 |
| 2.2128 | 948.71 | 1102.8 | 1504.2 | 1664 | 2492.6 | 4098.6 |
| 2.2212 | 846.64 | 1126.8 | 1426.4 | 1614.8 | 2382.5 | 4016.4 |
| 2.2296 | 861.52 | 1127.5 | 1440.8 | 1575.4 | 2370.1 | 3960.7 |
| 2.238 | 946.99 | 1112.8 | 1510.5 | 1541.8 | 2416.6 | 3920.7 |
| 2.2464 | 839.88 | 993.2 | 1396.5 | 1428 | 2269.3 | 3738.3 |
| 2.2548 | 778.74 | 914.08 | 1330.9 | 1392.1 | 2207.2 | 3629.2 |
| 2.2632 | 787.39 | 896.2 | 1339 | 1475.3 | 2275 | 3631.7 |
| 2.2715 | 749.92 | 842.46 | 1244.9 | 1418.8 | 2154.4 | 3511.8 |
| 2.2799 | 700.87 | 785.57 | 1128.9 | 1329 | 1997.9 | 3368.7 |
| 2.2883 | 686.55 | 815.24 | 1130.1 | 1370.1 | 2111.3 | 3396.9 |
| 2.2967 | 726.73 | 876.08 | 1181.4 | 1405.6 | 2184.1 | 3429.3 |
| 2.3051 | 845.55 | 982.31 | 1305.3 | 1433.7 | 2199.6 | 3468.1 |
| 2.3135 | 883.46 | 1023.5 | 1320.2 | 1464.7 | 2222.2 | 3487.1 |
| 2.3219 | 887.33 | 1037.3 | 1289.6 | 1496.6 | 2247.3 | 3497.4 |
| 2.3303 | 864.19 | 1023.3 | 1291.8 | 1505.5 | 2255.4 | 3457.1 |
| 2.3387 | 840.96 | 1000.4 | 1266.6 | 1486.5 | 2243.6 | 3395.1 |
| 2.3471 | 818.57 | 950.34 | 1149.9 | 1378.9 | 2168.9 | 3264.9 |
| 2.3555 | 798.26 | 923.91 | 1118.7 | 1320.6 | 2149.9 | 3217.1 |
| 2.3639 | 780.21 | 923.88 | 1183.2 | 1317.1 | 2193 | 3261.3 |
| 2.3723 | 815.67 | 951.09 | 1204.1 | 1359.6 | 2203.1 | 3289.9 |
| 2.3807 | 875.73 | 990.71 | 1204.4 | 1423 | 2197.6 | 3310.7 |
| 2.389 | 864.16 | 996.02 | 1207.6 | 1433.4 | 2151.8 | 3323.6 |
| 2.3974 | 840.2 | 993.8 | 1209 | 1432.9 | 2100.7 | 3331.4 |
| 2.4058 | 764.1 | 915.3 | 1136.4 | 1343.9 | 2066.4 | 3212.2 |
| 2.4142 | 709.95 | 856.84 | 1085.4 | 1282.6 | 2044.9 | 3119.1 |
| 2.4226 | 758.22 | 891.24 | 1135.6 | 1351.1 | 2084.7 | 3145.9 |
| 2.431 | 751.07 | 876.65 | 1126.6 | 1367.7 | 2062.8 | 3117.4 |
| 2.4394 | 607.29 | 741.05 | 971.41 | 1257.3 | 1888.1 | 2952.4 |
| 2.4478 | 551.53 | 682.51 | 908.64 | 1195.1 | 1829.3 | 2885.4 |
| 2.4562 | 637.98 | 748.37 | 995.16 | 1210.1 | 1957.8 | 2976.7 |
| 2.4646 | 675.64 | 794.47 | 1029.4 | 1225.9 | 1998.8 | 2995.3 |
| 2.473 | 653.35 | 816.47 | 999.43 | 1242.7 | 1932.2 | 2924.3 |
| 2.4814 | 622.59 | 811.94 | 975.3 | 1227.2 | 1877.8 | 2881.4 |
| 2.4897 | 582.92 | 779.59 | 957.31 | 1177.9 | 1836.1 | 2867.9 |
| 2.4981 | 617.42 | 795.52 | 987.26 | 1190.7 | 1871.6 | 2894.5 |
| 2.5065 | 725.35 | 859.25 | 1064.7 | 1264.9 | 1983.6 | 2960.7 |
| 2.5149 | 732.8 | 869.87 | 1064.1 | 1281.7 | 2003.3 | 2936.1 |
| 2.5233 | 635.73 | 825.27 | 982.46 | 1238.9 | 1926.9 | 2817.1 |
| 2.5317 | 620.98 | 800.1 | 956.88 | 1222 | 1916.1 | 2781.8 |
| 2.5401 | 706.66 | 798.55 | 999.62 | 1236.9 | 1985.4 | 2848.6 |
| 2.5485 | 702.6 | 797.77 | 1021.5 | 1239.8 | 1977.7 | 2888 |
| 2.5569 | 688.9 | 798.27 | 1010 | 1223.5 | 1845.6 | 2883.9 |
| 2.5653 | 658.2 | 812.69 | 1014.9 | 1226.7 | 1776.6 | 2888.5 |
| 2.5737 | 723.1 | 862.49 | 1061.4 | 1278.7 | 1863.6 | 2915 |
| 2.5821 | 731.59 | 881.25 | 1131.1 | 1293.4 | 1916 | 2911.5 |
| 2.5905 | 605.53 | 736.95 | 1330.3 | 1111.9 | 1789.5 | 2749.5 |

**2.4. Beam current-dependent CL spectra, non-dislocated, center (Fig. 7 (c))**

| **Photon energy (eV)** | **247 pA** | **302 pA** | **380 pA** | **452 pA** | **668 pA** | **1070 pA** |
| --- | --- | --- | --- | --- | --- | --- |
| 1.1973 | 496.66 | 702.95 | 421.36 | 518.63 | 431.33 | 300.6 |
| 1.2057 | 474.5 | 249.23 | 355.51 | 309.15 | 674.51 | 1074.7 |
| 1.2141 | 264.74 | 209.56 | 274.46 | 236.02 | 458.96 | 903.27 |
| 1.2225 | 417.23 | 447.43 | 403.5 | 590.95 | 779.02 | 1357.8 |
| 1.2309 | 16.721 | 62.591 | 107.01 | 263.72 | 375.94 | 1214.7 |
| 1.2393 | 237.66 | 151.72 | 263.95 | 417.99 | 763.81 | 1587 |
| 1.2477 | 1195.8 | 1172.4 | 1034.5 | 1302.3 | 1754.6 | 2590.5 |
| 1.2561 | 473.96 | 587.75 | 518.9 | 735.25 | 1264.3 | 2507.6 |
| 1.2645 | 960.7 | 947.5 | 995.5 | 1281 | 1791.6 | 3101.7 |
| 1.2729 | 423.53 | 698.16 | 715.9 | 736.85 | 1694.7 | 2924.2 |
| 1.2813 | 811.43 | 807.13 | 1063.4 | 1443.1 | 2262.9 | 3673.4 |
| 1.2896 | 803.59 | 1181.6 | 1205.1 | 1424.7 | 2679.4 | 3850.4 |
| 1.298 | 915.63 | 1060.8 | 1150 | 1161 | 2516.5 | 4660.2 |
| 1.3064 | 1141.3 | 1468.3 | 1473.3 | 1944.3 | 3498.2 | 4965.4 |
| 1.3148 | 953.21 | 1355 | 1438.3 | 1887.9 | 3384.3 | 5003.2 |
| 1.3232 | 1454.5 | 1859.8 | 2042.1 | 2396.8 | 4277.7 | 5960.6 |
| 1.3316 | 1037 | 1405.9 | 1738.1 | 2135.5 | 4104.3 | 6093.5 |
| 1.34 | 2374.9 | 2541.9 | 3064.6 | 3475.8 | 5534.1 | 7195.7 |
| 1.3484 | 1893.3 | 2293.9 | 2787.3 | 3396.4 | 5440 | 7467.4 |
| 1.3568 | 1921.7 | 2174.7 | 2634.3 | 3401.6 | 5407.7 | 7636.8 |
| 1.3652 | 2143 | 2603 | 3200.6 | 3906.3 | 6267.5 | 8086.6 |
| 1.3736 | 2673.1 | 2885.9 | 3598 | 4468 | 6661.2 | 8826.1 |
| 1.382 | 2431.1 | 2498.9 | 3464.2 | 4399.4 | 6708.1 | 8987.9 |
| 1.3903 | 3140.7 | 3258.2 | 3996.4 | 5228.5 | 7258 | 9824.8 |
| 1.3987 | 2977.7 | 3064.3 | 3617.7 | 4998.5 | 7189.7 | 10133 |
| 1.4071 | 2846.2 | 3134.9 | 3836.3 | 5145 | 7437 | 10856 |
| 1.4155 | 2993.8 | 3293.5 | 3925.6 | 5187.4 | 7422.1 | 11516 |
| 1.4239 | 3428.1 | 3664.6 | 4152.9 | 5642.8 | 7875.4 | 12496 |
| 1.4323 | 3128.8 | 3302.2 | 4008.6 | 5097 | 7546.5 | 12357 |
| 1.4407 | 3475 | 4013.2 | 4329.8 | 5627.2 | 7961.4 | 12868 |
| 1.4491 | 3601.1 | 3840.6 | 4432.6 | 5762.1 | 7863.2 | 12566 |
| 1.4575 | 3614.8 | 3856.4 | 4519.6 | 5563.8 | 7657.3 | 12556 |
| 1.4659 | 3385.7 | 3670.5 | 4272.9 | 5562.2 | 7601 | 11880 |
| 1.4743 | 3777.2 | 3883.4 | 4659.6 | 5840.9 | 7617.1 | 11958 |
| 1.4827 | 3521.9 | 3701.1 | 4120.1 | 5395.3 | 7216.5 | 11394 |
| 1.4911 | 3489.9 | 3776.3 | 4216.5 | 5597.2 | 7398.7 | 11298 |
| 1.4995 | 3454.8 | 3839.2 | 4237 | 5592.6 | 7316.1 | 11005 |
| 1.5078 | 3568.6 | 4024.2 | 4419 | 5761 | 7589.9 | 11494 |
| 1.5162 | 3732.4 | 4033 | 4175 | 5383.1 | 7666.4 | 11501 |
| 1.5246 | 3627.5 | 3938 | 4097.9 | 5151.3 | 7725.6 | 11496 |
| 1.533 | 3619.6 | 4200.9 | 4164.4 | 5366.1 | 7963.1 | 11734 |
| 1.5414 | 4183.7 | 4223.4 | 4542.4 | 5627.1 | 8428.5 | 12602 |
| 1.5498 | 3761.5 | 4392 | 4226.5 | 5240 | 8034.7 | 12554 |
| 1.5582 | 4036.5 | 4123.9 | 4630.1 | 5605.1 | 8274.1 | 13065 |
| 1.5666 | 4023.3 | 4287.4 | 4842.9 | 5689.2 | 8406.2 | 13522 |
| 1.575 | 3973.8 | 4221.7 | 4645.3 | 5571.8 | 8338.8 | 13422 |
| 1.5834 | 3605.8 | 4187.4 | 4656.1 | 5213 | 8159.6 | 13049 |
| 1.5918 | 3472.8 | 4062.5 | 4453.6 | 5197.9 | 7823.7 | 12823 |
| 1.6002 | 3147.5 | 3799.6 | 4119.4 | 4667 | 7566.4 | 12358 |
| 1.6086 | 3102.5 | 3924.6 | 4343.3 | 4651.3 | 7441.2 | 11913 |
| 1.6169 | 2941.9 | 3649 | 4285 | 4390 | 7084.4 | 11407 |
| 1.6253 | 3263.6 | 3847.8 | 4538.1 | 4439.6 | 7243.2 | 11368 |
| 1.6337 | 3034.8 | 3435.9 | 4510.7 | 4417 | 6894 | 10864 |
| 1.6421 | 2945.8 | 3451.9 | 4373.3 | 4155.2 | 6655.3 | 10388 |
| 1.6505 | 2801.5 | 3113.7 | 4345.8 | 4071.7 | 6444.7 | 10031 |
| 1.6589 | 2629 | 3185.2 | 4292.9 | 4066.1 | 6345.3 | 9854 |
| 1.6673 | 2432.2 | 3092.3 | 4164 | 3995.4 | 6327.1 | 9670.6 |
| 1.6757 | 2116.3 | 2729.9 | 3736.4 | 3710.2 | 5964.5 | 9166.3 |
| 1.6841 | 2264.7 | 2881.9 | 3759.8 | 3836.1 | 6005.3 | 9304.9 |
| 1.6925 | 2140.8 | 2702 | 3578.2 | 3560.1 | 5840.6 | 9078.3 |
| 1.7009 | 2398.2 | 2763.4 | 3814.6 | 3825.4 | 6036.7 | 9259.7 |
| 1.7093 | 2161.4 | 2600.4 | 3500.9 | 3532.1 | 5967.5 | 9023.6 |
| 1.7177 | 2351.8 | 2946.2 | 3901.9 | 3605.4 | 6058.9 | 9171.3 |
| 1.726 | 2371.4 | 2578.4 | 3713 | 3723.4 | 5969.7 | 9068.2 |
| 1.7344 | 2025.6 | 2242.8 | 3298.8 | 3096.8 | 5180.7 | 8232.3 |
| 1.7428 | 2201 | 2372 | 3626.7 | 3401 | 5268.4 | 8174.7 |
| 1.7512 | 2043.9 | 2331 | 3292.2 | 3300.8 | 5068.2 | 7883.4 |
| 1.7596 | 1914 | 2172.7 | 3128.8 | 3227.3 | 4623.8 | 7591.5 |
| 1.768 | 2029.9 | 2245.1 | 3286.9 | 3423.5 | 4720.4 | 7677.2 |
| 1.7764 | 1957.8 | 2329.1 | 3143.9 | 3422.8 | 4406.2 | 7504.9 |
| 1.7848 | 2368.1 | 2378.5 | 3366.3 | 3674.3 | 4832.4 | 7481.6 |
| 1.7932 | 2080.7 | 2206.4 | 2985.4 | 3436.3 | 4345.9 | 7177.5 |
| 1.8016 | 2321.9 | 2321.4 | 3043.5 | 3554.2 | 4391.2 | 7472.8 |
| 1.81 | 1935.2 | 2207.4 | 2791.9 | 3358.6 | 4240.5 | 7425.2 |
| 1.8184 | 2211.2 | 2252.6 | 2743.4 | 3284.5 | 4132.3 | 7350.8 |
| 1.8268 | 2160.7 | 2257.2 | 2756.9 | 3245 | 4229.3 | 7530.1 |
| 1.8351 | 1895.4 | 2120.8 | 2415.5 | 3015.3 | 4091.4 | 7490.9 |
| 1.8435 | 1893.9 | 2248.5 | 2511.6 | 3036.2 | 4109.5 | 7732.9 |
| 1.8519 | 1810.3 | 2110 | 2447.6 | 2936.8 | 4089.2 | 7739.8 |
| 1.8603 | 1904.8 | 2118.4 | 2431.3 | 2914.1 | 4192.1 | 7797.1 |
| 1.8687 | 2128.5 | 2354.6 | 2563.2 | 3057.5 | 4417.6 | 8007.3 |
| 1.8771 | 2126.2 | 2337 | 2547.7 | 3067.6 | 4496.1 | 7994.2 |
| 1.8855 | 2102.9 | 2177.3 | 2531.5 | 2895.8 | 4436.4 | 7812.3 |
| 1.8939 | 1942.6 | 2257 | 2362.4 | 2834.9 | 4398 | 7725.5 |
| 1.9023 | 2043.9 | 2281.5 | 2643.8 | 2830 | 4541.6 | 7753.4 |
| 1.9107 | 2123.1 | 2405 | 2709.7 | 2883 | 4652.9 | 7711.4 |
| 1.9191 | 2013.4 | 2297 | 2638.6 | 2764.5 | 4547.6 | 7416.5 |
| 1.9275 | 2022.2 | 2253.8 | 2657.4 | 2770.7 | 4519.3 | 7133.2 |
| 1.9359 | 2156.7 | 2356.8 | 2748.3 | 2927.9 | 4650.4 | 6980.5 |
| 1.9442 | 1908.4 | 2182.8 | 2579.6 | 2746.6 | 4580.7 | 6713.4 |
| 1.9526 | 1967.1 | 2268.5 | 2610.4 | 2775.9 | 4609.4 | 6459.1 |
| 1.961 | 1814.8 | 2152 | 2533.2 | 2530.7 | 4262.5 | 6164.5 |
| 1.9694 | 1782.9 | 2048 | 2477.2 | 2405.6 | 4070.9 | 5930.2 |
| 1.9778 | 1812.2 | 1992.6 | 2441 | 2426.9 | 4048.4 | 5773.1 |
| 1.9862 | 1724.3 | 1945.2 | 2380 | 2468.5 | 4014.8 | 5661.9 |
| 1.9946 | 1642.7 | 1799 | 2335.6 | 2312.4 | 3878.4 | 5398.6 |
| 2.003 | 1598.9 | 1743 | 2316.3 | 2259.8 | 3845.1 | 5185.1 |
| 2.0114 | 1558.7 | 1726.7 | 2275.5 | 2220.3 | 3639.8 | 5026.4 |
| 2.0198 | 1506.4 | 1730.5 | 2212.2 | 2174.5 | 3311.5 | 4912.5 |
| 2.0282 | 1431 | 1753.7 | 2174.4 | 2164 | 3308.9 | 4812.5 |
| 2.0366 | 1416.4 | 1569.5 | 2046.7 | 2074.3 | 3215.6 | 4647.9 |
| 2.045 | 1431.7 | 1480.1 | 2039.9 | 2027.7 | 3124.9 | 4532.8 |
| 2.0533 | 1449.4 | 1498 | 2110 | 2016 | 3024.9 | 4436.9 |
| 2.0617 | 1401.5 | 1517.7 | 2019.3 | 1953.5 | 2825.4 | 4206.2 |
| 2.0701 | 1258.1 | 1378.9 | 1827.7 | 1815.5 | 2643 | 4024.9 |
| 2.0785 | 1127.8 | 1242.5 | 1669.3 | 1715.3 | 2449.7 | 3863.2 |
| 2.0869 | 1045 | 1156.6 | 1583.4 | 1682.6 | 2249 | 3717.8 |
| 2.0953 | 1086.6 | 1191 | 1604.9 | 1679.3 | 2261.9 | 3675.7 |
| 2.1037 | 1115.2 | 1212.7 | 1529.2 | 1622.9 | 2235 | 3589.7 |
| 2.1121 | 1124.2 | 1207.5 | 1434.4 | 1560.4 | 2191.5 | 3480 |
| 2.1205 | 1088.4 | 1129.7 | 1464.8 | 1582.1 | 2167.1 | 3370.5 |
| 2.1289 | 849.44 | 948.91 | 1186.7 | 1324.5 | 1866.1 | 3104.9 |
| 2.1373 | 696.4 | 839.69 | 987.24 | 1141.2 | 1656.5 | 2895.5 |
| 2.1457 | 856.56 | 949.68 | 1151.7 | 1293.9 | 1818 | 2905 |
| 2.1541 | 753.46 | 876.18 | 1041.3 | 1204.7 | 1759.9 | 2843.5 |
| 2.1624 | 654.56 | 811.26 | 947.11 | 1118.1 | 1681.7 | 2772.5 |
| 2.1708 | 658.47 | 828.62 | 982.82 | 1123.9 | 1645.2 | 2709.2 |
| 2.1792 | 616.9 | 766.82 | 939.54 | 1094.8 | 1622.5 | 2499.2 |
| 2.1876 | 644.33 | 762 | 934.43 | 1092.6 | 1650.2 | 2379.8 |
| 2.196 | 846.34 | 919.38 | 1049.5 | 1165.6 | 1784.5 | 2525.7 |
| 2.2044 | 836.8 | 911.91 | 1052.2 | 1186.6 | 1744.1 | 2512.6 |
| 2.2128 | 766.78 | 858.53 | 1015.8 | 1179.7 | 1656.7 | 2449 |
| 2.2212 | 718.6 | 827.21 | 962.36 | 1126.1 | 1598.8 | 2381.1 |
| 2.2296 | 747.03 | 864.03 | 965.99 | 1116.5 | 1614.1 | 2391.6 |
| 2.238 | 820.94 | 940.94 | 1003.8 | 1133.1 | 1673.1 | 2448.5 |
| 2.2464 | 694.51 | 808.3 | 909.47 | 1017.5 | 1571.8 | 2283.6 |
| 2.2548 | 635.09 | 729.32 | 867.77 | 968.15 | 1526.7 | 2182.8 |
| 2.2632 | 677.81 | 731.7 | 906.3 | 1019.8 | 1567.2 | 2179.3 |
| 2.2715 | 609.24 | 682.22 | 838.46 | 966.62 | 1547.6 | 2114.3 |
| 2.2799 | 515.06 | 622.4 | 747.77 | 887.7 | 1508.7 | 2041.4 |
| 2.2883 | 532.25 | 629.58 | 780.71 | 898.95 | 1475.4 | 2094.9 |
| 2.2967 | 573.6 | 680.82 | 845.96 | 963.46 | 1486.7 | 2163.8 |
| 2.3051 | 650.07 | 795.85 | 958.26 | 1105.1 | 1562.2 | 2255.8 |
| 2.3135 | 692.69 | 825.91 | 971.17 | 1135.9 | 1630.8 | 2305.3 |
| 2.3219 | 720.72 | 820.36 | 942.69 | 1120.4 | 1695.6 | 2336.5 |
| 2.3303 | 747.88 | 843.93 | 921.8 | 1135.4 | 1705.5 | 2280.9 |
| 2.3387 | 750.61 | 846.17 | 892.21 | 1128.4 | 1690 | 2217.8 |
| 2.3471 | 675.1 | 777.81 | 832.77 | 1047.9 | 1596.7 | 2134.6 |
| 2.3555 | 657.03 | 749.79 | 832.31 | 1027.6 | 1570.4 | 2110 |
| 2.3639 | 703.16 | 766.8 | 897.79 | 1074.6 | 1619.1 | 2151.1 |
| 2.3723 | 735.02 | 801.79 | 911.77 | 1098.9 | 1642 | 2200.3 |
| 2.3807 | 759.82 | 844.78 | 901.46 | 1112.4 | 1652.4 | 2252.6 |
| 2.389 | 749.62 | 827.99 | 927.12 | 1081.6 | 1592.6 | 2232.7 |
| 2.3974 | 731.39 | 801.06 | 954.73 | 1043.5 | 1521.9 | 2200.7 |
| 2.4058 | 611.94 | 733.84 | 865.72 | 972.86 | 1421.6 | 2129.8 |
| 2.4142 | 526.3 | 686.8 | 800.02 | 923.71 | 1353.6 | 2072 |
| 2.4226 | 598.13 | 734.18 | 842.46 | 974.96 | 1437.2 | 2074.1 |
| 2.431 | 674.55 | 725.49 | 832.13 | 973.39 | 1474 | 2049.8 |
| 2.4394 | 764.74 | 578.52 | 691.65 | 841.76 | 1396.9 | 1960 |
| 2.4478 | 772.3 | 515.81 | 643.89 | 792.4 | 1374.3 | 1926.4 |
| 2.4562 | 645.85 | 589.1 | 746.07 | 875.97 | 1440 | 1983.4 |
| 2.4646 | 565.26 | 630.25 | 782.95 | 912.64 | 1464.7 | 2001.9 |
| 2.473 | 540.82 | 631.97 | 739.47 | 891.64 | 1438.9 | 1972.8 |
| 2.4814 | 511.96 | 610.42 | 706.65 | 863.23 | 1380.4 | 1949.6 |
| 2.4897 | 478.41 | 564.44 | 684.94 | 827.03 | 1287.6 | 1932.5 |
| 2.4981 | 520.22 | 584.7 | 701.71 | 841.26 | 1283.4 | 1975 |
| 2.5065 | 636.65 | 670.52 | 756.56 | 905.42 | 1366.7 | 2076.6 |
| 2.5149 | 650.57 | 678.85 | 750.24 | 922.66 | 1385.3 | 2064.1 |
| 2.5233 | 557.87 | 606.55 | 680.28 | 891.11 | 1336.6 | 1932.9 |
| 2.5317 | 532.56 | 591.59 | 671.34 | 889.14 | 1318.9 | 1891.3 |
| 2.5401 | 589.39 | 646.54 | 736.87 | 923.28 | 1339 | 1959 |
| 2.5485 | 505.56 | 670.95 | 762.22 | 922.1 | 1331.8 | 1978 |
| 2.5569 | 644.7 | 646.38 | 723.03 | 863.89 | 1280.5 | 1918.6 |
| 2.5653 | 569.1 | 638.23 | 714.51 | 835.25 | 1267.5 | 1883.7 |
| 2.5737 | 465.6 | 671 | 782.58 | 879.81 | 1350.2 | 1909.2 |
| 2.5821 | 654.9 | 682.4 | 811.32 | 894.56 | 1392.4 | 1909.1 |
| 2.5905 | 485.85 | 581.44 | 633.76 | 752.38 | 1223.6 | 1773.4 |

**2.5. Beam current-dependent CL spectra, dislocated, edge (Fig. 7 (e))**

| **Photon energy (eV)** | **247 pA** | **302 pA** | **380 pA** | **450 pA** | **668 pA** | **1070 pA** |
| --- | --- | --- | --- | --- | --- | --- |
| 1.1973 | 496.12 | 641.69 | 376.12 | 499.39 | 342.2 | 78.515 |
| 1.2057 | 368.11 | 300.84 | 680.55 | 670.81 | 908.29 | 1094.3 |
| 1.2141 | 291.39 | 457 | 533.43 | 452.55 | 524.9 | 1026 |
| 1.2225 | 616.95 | 610.25 | 812.33 | 899.26 | 1354.9 | 1606.9 |
| 1.2309 | 112.47 | 245.23 | 346.39 | 398.88 | 850.96 | 1390.3 |
| 1.2393 | 341.75 | 491.39 | 490.19 | 692.76 | 1302.9 | 1923.7 |
| 1.2477 | 1233.4 | 1277.4 | 1676.2 | 1844.2 | 2400.6 | 3286.5 |
| 1.2561 | 658.5 | 823.2 | 1227.1 | 1402.6 | 2009.9 | 3079.8 |
| 1.2645 | 1063.7 | 1389 | 1395.4 | 2015.3 | 2594.1 | 3738.4 |
| 1.2729 | 887.1 | 1172.8 | 1323.3 | 1796.4 | 2592.9 | 4146.1 |
| 1.2813 | 1175.3 | 1533.9 | 2002.7 | 2197.8 | 3224.8 | 5233.5 |
| 1.2896 | 1496.8 | 1680.6 | 2238.1 | 2504 | 3970.6 | 6104.4 |
| 1.298 | 1421.9 | 1660.7 | 2255.5 | 2549 | 3893.8 | 6845.2 |
| 1.3064 | 2055.4 | 2218.1 | 2850.8 | 3267.5 | 4627.1 | 8022.5 |
| 1.3148 | 1824.7 | 2306.9 | 2967.2 | 3338.7 | 4905.2 | 8522.6 |
| 1.3232 | 2787.2 | 2862.5 | 3672.2 | 4083.7 | 6158.5 | 9895 |
| 1.3316 | 2302.3 | 2866.5 | 3468.9 | 4290.2 | 6342.2 | 10386 |
| 1.34 | 3527.8 | 4066.8 | 4942.2 | 5764.4 | 7827.1 | 12263 |
| 1.3484 | 3428.6 | 3924.7 | 4850.1 | 5738.8 | 7963.4 | 12115 |
| 1.3568 | 3247.7 | 3853.3 | 4669.9 | 5775.4 | 8237.7 | 12586 |
| 1.3652 | 3573.3 | 4155.3 | 5179.4 | 6646.5 | 9048.7 | 13375 |
| 1.3736 | 3989.6 | 4603.8 | 5589.6 | 7025.6 | 9671.2 | 14265 |
| 1.382 | 3762.3 | 4461.2 | 5277.6 | 6961.6 | 9562.9 | 13706 |
| 1.3903 | 4331.6 | 4996.9 | 6080.3 | 7596.3 | 10371 | 14358 |
| 1.3987 | 3919.7 | 4655.4 | 5580.2 | 7374.9 | 10296 | 14001 |
| 1.4071 | 3982.7 | 4697 | 5655.6 | 7306 | 10248 | 14034 |
| 1.4155 | 4139.1 | 4688.3 | 5638.9 | 7753.2 | 10638 | 14405 |
| 1.4239 | 4289.1 | 5021.9 | 5987.1 | 8147.8 | 10997 | 14504 |
| 1.4323 | 4070.9 | 4811 | 5632.9 | 8029.1 | 10726 | 14289 |
| 1.4407 | 4597.5 | 5157.3 | 6372.6 | 8711.5 | 11225 | 15159 |
| 1.4491 | 4527.5 | 5378.1 | 6189.8 | 8583.4 | 11610 | 15367 |
| 1.4575 | 4612.6 | 5236.8 | 6433.3 | 8742.8 | 11733 | 15681 |
| 1.4659 | 4204.4 | 5144.3 | 6264.1 | 8635.4 | 11791 | 15989 |
| 1.4743 | 4832.5 | 5451.6 | 6606.5 | 8860.8 | 12192 | 16572 |
| 1.4827 | 4516.6 | 5220.6 | 6527.6 | 8292.8 | 11932 | 16315 |
| 1.4911 | 4830.8 | 5497.2 | 6721.8 | 8420 | 11978 | 16894 |
| 1.4995 | 4820.8 | 5581.5 | 6802.6 | 8186.4 | 12021 | 17352 |
| 1.5078 | 5140.7 | 5964.9 | 7081.3 | 8491.8 | 12314 | 18068 |
| 1.5162 | 5024.4 | 5796.1 | 7139 | 8295 | 12251 | 18130 |
| 1.5246 | 4873.9 | 5649.9 | 7105.1 | 7884 | 12000 | 18081 |
| 1.533 | 4957.5 | 5671.6 | 6916.6 | 7569.5 | 12075 | 17806 |
| 1.5414 | 5164.7 | 5995 | 7163.9 | 7887 | 12376 | 17948 |
| 1.5498 | 4529.6 | 5275.4 | 6564.6 | 7399.5 | 11621 | 17521 |
| 1.5582 | 4567.6 | 5211.8 | 6560.5 | 7303.1 | 11624 | 17210 |
| 1.5666 | 4291.8 | 5035.6 | 6302.9 | 7265.4 | 11634 | 17100 |
| 1.575 | 4090.1 | 4681.6 | 6032 | 7034 | 11147 | 16841 |
| 1.5834 | 3873 | 4322.1 | 5610.8 | 6730.9 | 10603 | 16581 |
| 1.5918 | 3688.3 | 4328 | 5488.2 | 6495.5 | 10157 | 16431 |
| 1.6002 | 3081.5 | 3803.6 | 5273.2 | 5941.9 | 9859 | 16026 |
| 1.6086 | 3211.6 | 3963.3 | 5167.4 | 6129.7 | 10613 | 16106 |
| 1.6169 | 3075.6 | 3755.3 | 5045.2 | 5875.5 | 10171 | 15815 |
| 1.6253 | 3257.4 | 4050.6 | 5422.4 | 6116.6 | 9583.6 | 16337 |
| 1.6337 | 3190.9 | 3816.6 | 5346.8 | 5858.5 | 9293.7 | 15951 |
| 1.6421 | 3264.1 | 3886.3 | 5304.9 | 5996.7 | 9314.2 | 15755 |
| 1.6505 | 3183.4 | 3733.8 | 5009.6 | 5759.3 | 8955.6 | 15433 |
| 1.6589 | 3038.5 | 3717.7 | 5024.5 | 5556.9 | 8727.2 | 15334 |
| 1.6673 | 2957.7 | 3640 | 4949.2 | 5608.3 | 8585.4 | 15102 |
| 1.6757 | 2615.7 | 3263.3 | 4549.6 | 5363.9 | 8354.2 | 14219 |
| 1.6841 | 2811.6 | 3452.1 | 4491.7 | 5266.4 | 8593.6 | 14033 |
| 1.6925 | 2675.7 | 3421 | 4430 | 5314 | 8531.4 | 13706 |
| 1.7009 | 2941.5 | 3677.2 | 4584.9 | 5634.4 | 8839.5 | 13644 |
| 1.7093 | 2871.3 | 3495.2 | 4486.8 | 5516.3 | 8715.6 | 13091 |
| 1.7177 | 3224.2 | 3642.3 | 4759.1 | 5734.7 | 8728.1 | 13274 |
| 1.726 | 2975.3 | 3631.5 | 4725.8 | 5892.2 | 8791.5 | 13423 |
| 1.7344 | 2489.9 | 3176.2 | 4238.1 | 5380.7 | 8325.4 | 12730 |
| 1.7428 | 2752.7 | 3510.4 | 4359.7 | 5599.6 | 8438.5 | 12782 |
| 1.7512 | 2802.2 | 3393.9 | 4379.2 | 5422.6 | 8327.9 | 12595 |
| 1.7596 | 2675.9 | 3163.3 | 4154.7 | 5533.2 | 8141.7 | 12426 |
| 1.768 | 2844.8 | 3296.6 | 4295.8 | 5443.7 | 8243.2 | 12642 |
| 1.7764 | 2788.8 | 3370.2 | 4365.3 | 5533.9 | 8150.2 | 12720 |
| 1.7848 | 3041.2 | 3613.2 | 4530.2 | 5821.1 | 8234.4 | 13175 |
| 1.7932 | 2891.9 | 3505.4 | 4187.4 | 5434.6 | 7837.1 | 12803 |
| 1.8016 | 2910.2 | 3595.9 | 4738.4 | 5712.3 | 7857.4 | 13049 |
| 1.81 | 2972.1 | 3328 | 4399.3 | 5466.3 | 7740.5 | 12740 |
| 1.8184 | 3022.5 | 3575.5 | 4687.9 | 5622.8 | 8015.8 | 12807 |
| 1.8268 | 3183.6 | 3652.2 | 4535.5 | 5513.2 | 7937.6 | 13003 |
| 1.8351 | 3034.8 | 3493.5 | 4478.3 | 5245 | 7668.9 | 12612 |
| 1.8435 | 3082.2 | 3641.6 | 4579.1 | 5336.2 | 7824.8 | 12530 |
| 1.8519 | 3011.5 | 3686.9 | 4482.8 | 5353.2 | 7859.3 | 12245 |
| 1.8603 | 3022.3 | 3727.2 | 4560.9 | 5449.3 | 8050.2 | 12008 |
| 1.8687 | 3225.1 | 3843.4 | 4764.7 | 5690.9 | 8410.1 | 11973 |
| 1.8771 | 3308.9 | 3827.7 | 4649.1 | 5744.6 | 8487.5 | 11806 |
| 1.8855 | 3131.3 | 3701.5 | 4398 | 5541.2 | 8380.4 | 11508 |
| 1.8939 | 3012 | 3675.3 | 4293 | 5470.5 | 8158.4 | 11135 |
| 1.9023 | 3009 | 3662.7 | 4300 | 5433.7 | 8330.6 | 11072 |
| 1.9107 | 3034.1 | 3667.8 | 4507.6 | 5438 | 8464.4 | 11155 |
| 1.9191 | 2836.1 | 3530.4 | 4340.5 | 5221.2 | 8282 | 11029 |
| 1.9275 | 2768.7 | 3434.4 | 4156 | 5069 | 8049.9 | 10914 |
| 1.9359 | 2902.3 | 3418.3 | 4170.1 | 5035.4 | 7863.7 | 10888 |
| 1.9442 | 2733 | 3219.8 | 4017.6 | 4693.1 | 7406.9 | 10622 |
| 1.9526 | 2670.2 | 3262.5 | 3892.4 | 4664.3 | 7232.4 | 10427 |
| 1.961 | 2586.8 | 2952.8 | 3678.8 | 4220.5 | 6899.2 | 10147 |
| 1.9694 | 2387.7 | 2787.1 | 3476.8 | 3921.1 | 6692.2 | 9850.9 |
| 1.9778 | 2147.8 | 2733.6 | 3318 | 3790.5 | 6474.3 | 9540.6 |
| 1.9862 | 1992.5 | 2585.3 | 3202.3 | 3648.9 | 6014.9 | 9208.1 |
| 1.9946 | 1929.2 | 2435.1 | 3105.6 | 3363.7 | 5756.3 | 8998.2 |
| 2.003 | 1857.3 | 2358.9 | 2889.7 | 3234.3 | 5437.5 | 8583.8 |
| 2.0114 | 1770.7 | 2250.9 | 2757.3 | 3081.1 | 5163.2 | 8188.1 |
| 2.0198 | 1678.1 | 2099.5 | 2729.5 | 2881.8 | 4934.5 | 7902.6 |
| 2.0282 | 1612.6 | 1982.6 | 2653.7 | 2765.8 | 4640.1 | 7769.9 |
| 2.0366 | 1541.6 | 1908.3 | 2464.1 | 2612.7 | 4386.2 | 7333.6 |
| 2.045 | 1466 | 1836.1 | 2379.5 | 2493.8 | 4228.4 | 6974.2 |
| 2.0533 | 1403.4 | 1772.9 | 2371 | 2412.5 | 4114.4 | 6730.7 |
| 2.0617 | 1410.8 | 1763.4 | 2244.5 | 2346.7 | 3858.5 | 6538.7 |
| 2.0701 | 1269 | 1589.1 | 2069.3 | 2156.2 | 3618.2 | 6184 |
| 2.0785 | 1149.3 | 1426.3 | 1919.4 | 2006.3 | 3390.7 | 5920 |
| 2.0869 | 1099.5 | 1327.9 | 1819.6 | 1940 | 3180.2 | 5807.1 |
| 2.0953 | 1123.9 | 1370 | 1847 | 1903.9 | 3078.9 | 5643 |
| 2.1037 | 1110.8 | 1336.4 | 1793.6 | 1891.8 | 3061.3 | 5468.5 |
| 2.1121 | 1091.6 | 1272.3 | 1720.5 | 1886 | 3057.7 | 5308.1 |
| 2.1205 | 1126.2 | 1249.6 | 1737.7 | 1854.8 | 2936.4 | 5218.9 |
| 2.1289 | 869.96 | 1082.8 | 1534.2 | 1605.3 | 2697.3 | 4967.1 |
| 2.1373 | 685.19 | 973.37 | 1392.7 | 1425.5 | 2540.6 | 4830.7 |
| 2.1457 | 837.93 | 1078.7 | 1523.8 | 1533.5 | 2634.8 | 5045.9 |
| 2.1541 | 814.39 | 1048.1 | 1453.8 | 1526.8 | 2556.4 | 4762.4 |
| 2.1624 | 788.36 | 1025 | 1387.6 | 1497.8 | 2479.5 | 4494.3 |
| 2.1708 | 821.75 | 1065.5 | 1401.3 | 1468.8 | 2467.9 | 4434 |
| 2.1792 | 781.24 | 1025.9 | 1374.3 | 1539.5 | 2418 | 4349.6 |
| 2.1876 | 799.18 | 1009.6 | 1374.4 | 1604 | 2425.9 | 4337.4 |
| 2.196 | 980.73 | 1080.4 | 1453.3 | 1611.8 | 2579.9 | 4496.7 |
| 2.2044 | 962.78 | 1108.1 | 1474.6 | 1574.8 | 2533.2 | 4494.2 |
| 2.2128 | 905.62 | 1126.1 | 1454.5 | 1537 | 2453.3 | 4441.7 |
| 2.2212 | 941.95 | 1160.7 | 1339.5 | 1551.8 | 2491.9 | 4391.5 |
| 2.2296 | 948.18 | 1162.2 | 1316.2 | 1567 | 2494.7 | 4364.6 |
| 2.238 | 935.45 | 1142.8 | 1349.2 | 1581.7 | 2475 | 4351.1 |
| 2.2464 | 888.76 | 1073.4 | 1336.2 | 1506.7 | 2421.9 | 4204.7 |
| 2.2548 | 866.39 | 1061 | 1341.5 | 1478.4 | 2372.1 | 4137.3 |
| 2.2632 | 881.08 | 1136.1 | 1374.9 | 1521.5 | 2326.9 | 4190.5 |
| 2.2715 | 804.87 | 1086.9 | 1341.5 | 1507.6 | 2310.9 | 4092.7 |
| 2.2799 | 712.67 | 1000.5 | 1288.9 | 1475.7 | 2304.4 | 3955.1 |
| 2.2883 | 761.72 | 949.35 | 1264.6 | 1452.2 | 2294 | 3916 |
| 2.2967 | 829.2 | 989.01 | 1290.3 | 1508.1 | 2334.4 | 3975.3 |
| 2.3051 | 923.84 | 1159.5 | 1388 | 1678.5 | 2448.2 | 4176.7 |
| 2.3135 | 962.84 | 1181.1 | 1422.2 | 1700.4 | 2475 | 4164.7 |
| 2.3219 | 978.25 | 1140.8 | 1429.8 | 1660.4 | 2465.2 | 4064.2 |
| 2.3303 | 1008.2 | 1172.2 | 1465.9 | 1722.2 | 2516 | 4072 |
| 2.3387 | 1009.4 | 1175.6 | 1469.8 | 1747 | 2526.5 | 4066.8 |
| 2.3471 | 916.8 | 1084.1 | 1367.7 | 1646.2 | 2404.1 | 4012 |
| 2.3555 | 890.79 | 1018.5 | 1324.6 | 1603.4 | 2344 | 3993.2 |
| 2.3639 | 939.13 | 981.45 | 1347.4 | 1625.4 | 2353.3 | 4014.8 |
| 2.3723 | 948.8 | 1016.4 | 1415.1 | 1645.7 | 2395.1 | 4030 |
| 2.3807 | 940.25 | 1084.5 | 1503.2 | 1665 | 2451.4 | 4042 |
| 2.389 | 965.64 | 1116.6 | 1494.7 | 1663.8 | 2423 | 3985.1 |
| 2.3974 | 992.06 | 1139 | 1469.1 | 1657.2 | 2381.4 | 3917 |
| 2.4058 | 879.41 | 1032.1 | 1358.3 | 1574.9 | 2327.4 | 3806.2 |
| 2.4142 | 796.73 | 952.44 | 1271.7 | 1508.7 | 2290.2 | 3733 |
| 2.4226 | 853.14 | 998.76 | 1295.8 | 1515.8 | 2332.2 | 3837.1 |
| 2.431 | 855.82 | 1010.6 | 1270.4 | 1503.1 | 2324.9 | 3867 |
| 2.4394 | 726.33 | 938.03 | 1122.2 | 1441.8 | 2196.2 | 3715.6 |
| 2.4478 | 687.28 | 915.9 | 1086.8 | 1412.7 | 2144.5 | 3626.8 |
| 2.4562 | 794.56 | 975.43 | 1233.9 | 1435.7 | 2217.3 | 3638.7 |
| 2.4646 | 836.28 | 993.46 | 1295.4 | 1594 | 2242.1 | 3623.9 |
| 2.473 | 797.36 | 960.42 | 1251.8 | 1736.3 | 2208 | 3576 |
| 2.4814 | 758.13 | 919.96 | 1205.4 | 1529.1 | 2177.5 | 3508.3 |
| 2.4897 | 718.52 | 871.66 | 1156 | 1415.1 | 2150.8 | 3419.7 |
| 2.4981 | 750.5 | 889.02 | 1177.2 | 1354.4 | 2160.5 | 3447.7 |
| 2.5065 | 853.34 | 971.37 | 1268.2 | 1493.5 | 2206.5 | 3591.4 |
| 2.5149 | 851.77 | 982.74 | 1281.6 | 1518.4 | 2189.7 | 3608.6 |
| 2.5233 | 741.6 | 920.26 | 1214.4 | 1424.6 | 2107.5 | 3494.5 |
| 2.5317 | 706.23 | 909.38 | 1199.5 | 1392.6 | 2086.2 | 3435.6 |
| 2.5401 | 762 | 961.43 | 1248.7 | 1435.8 | 2139.1 | 3443.9 |
| 2.5485 | 792.27 | 978.44 | 1259.2 | 1447.2 | 2143.4 | 3443.6 |
| 2.5569 | 781.79 | 939.09 | 1207.5 | 1407.4 | 2069.2 | 3429.5 |
| 2.5653 | 791 | 932.74 | 1189.8 | 1394.5 | 2033.9 | 3422.4 |
| 2.5737 | 849.83 | 1008.9 | 1257.1 | 1448.5 | 2094.8 | 3432.7 |
| 2.5821 | 875.86 | 1043.5 | 1289.5 | 1465.1 | 2122.7 | 3410.2 |
| 2.5905 | 730.03 | 860.27 | 1139.8 | 1285.1 | 1978.2 | 3211.6 |

**2.6. Beam current-dependent CL spectra, dislocated, center (Fig. 7 (f))**

| **Photon energy (eV)** | **247 pA** | **302 pA** | **380 pA** | **450 pA** | **668 pA** | **1070 pA** |
| --- | --- | --- | --- | --- | --- | --- |
| 1.1973 | 507.93 | 727.2 | 399.57 | 537.55 | 700 | 651 |
| 1.2057 | 296.6 | 530.58 | 490.56 | 656.25 | 706.9 | 1146.1 |
| 1.2141 | 158.93 | 221.69 | 518.19 | 608.89 | 579.43 | 855.04 |
| 1.2225 | 524 | 813.86 | 577.75 | 841.13 | 1157.1 | 1545.6 |
| 1.2309 | 186.44 | 255.34 | 434.23 | 514.71 | 761.47 | 1243.9 |
| 1.2393 | 288.93 | 530 | 484.38 | 702.69 | 1245 | 2062.6 |
| 1.2477 | 1211.5 | 1419.5 | 1491.7 | 1847.4 | 2302 | 3077.3 |
| 1.2561 | 591.9 | 764.34 | 1157.4 | 1263.9 | 1804.7 | 2992.8 |
| 1.2645 | 1138.1 | 1456.3 | 1575.5 | 2087.9 | 2443.4 | 3857.7 |
| 1.2729 | 896.75 | 1239.9 | 1451.9 | 1630.4 | 2372.4 | 4332.4 |
| 1.2813 | 1208.3 | 1650.9 | 1986.6 | 2288.5 | 3134.8 | 5562.8 |
| 1.2896 | 1387.4 | 1803.7 | 2162.8 | 2534.2 | 3924.2 | 6512.6 |
| 1.298 | 1372.7 | 1875.7 | 2536.2 | 2681.9 | 3951.8 | 7528.7 |
| 1.3064 | 2177 | 2462.5 | 3155.7 | 3402.1 | 4842.3 | 8633.3 |
| 1.3148 | 1955.3 | 2400.8 | 3054.9 | 3352.7 | 5064.3 | 9471.9 |
| 1.3232 | 2760.1 | 3190.7 | 3938.6 | 4226.7 | 6278.7 | 11076 |
| 1.3316 | 2395.9 | 3182.2 | 3926.8 | 4534.2 | 6523.4 | 11566 |
| 1.34 | 3874.7 | 4594.6 | 5210 | 5977.3 | 8120.7 | 13449 |
| 1.3484 | 3410.1 | 4377.4 | 5370.4 | 6176.3 | 8213.2 | 13352 |
| 1.3568 | 3360.3 | 4353.3 | 5015.2 | 6098.5 | 8570.2 | 14095 |
| 1.3652 | 3953.1 | 4597.4 | 5682.6 | 6909.2 | 9324.4 | 14402 |
| 1.3736 | 4257.6 | 5129.2 | 6056.8 | 7433.8 | 9872.1 | 14884 |
| 1.382 | 4046.7 | 4616.3 | 5833.1 | 7390.1 | 9718.3 | 14436 |
| 1.3903 | 4556.6 | 5272.2 | 6396.2 | 8088.1 | 10050 | 14762 |
| 1.3987 | 4023 | 4704.8 | 5856.6 | 7756.8 | 10111 | 14354 |
| 1.4071 | 4029.2 | 4651.8 | 5612.8 | 7758.4 | 10138 | 13826 |
| 1.4155 | 3892.1 | 4723.6 | 5932.7 | 8107.8 | 10520 | 13977 |
| 1.4239 | 4147.5 | 5205.9 | 6078 | 8300.5 | 10566 | 13969 |
| 1.4323 | 3923.7 | 4706.4 | 5700.4 | 8125.6 | 10179 | 13649 |
| 1.4407 | 4346.8 | 5141.2 | 6262.9 | 8575.1 | 10767 | 14375 |
| 1.4491 | 4368.8 | 5210.5 | 6295.2 | 8320.9 | 10895 | 14315 |
| 1.4575 | 4333.5 | 5126.2 | 6325 | 8496.4 | 10838 | 14708 |
| 1.4659 | 3960 | 4946.2 | 6266.6 | 8115.9 | 10656 | 14800 |
| 1.4743 | 4405.4 | 5316.7 | 6490.3 | 8361.8 | 11138 | 15376 |
| 1.4827 | 4145.4 | 4876.9 | 6034.4 | 7568.9 | 10830 | 15307 |
| 1.4911 | 4320.1 | 5053.8 | 6417.6 | 7949.2 | 10769 | 15333 |
| 1.4995 | 4191.4 | 5125.9 | 6257.8 | 7552.8 | 10689 | 15599 |
| 1.5078 | 4583.1 | 5214.4 | 6604.8 | 7995.7 | 10842 | 15915 |
| 1.5162 | 4380.2 | 5032.6 | 6362 | 7639.3 | 10865 | 15815 |
| 1.5246 | 4060.7 | 4856.3 | 6115.6 | 7302.1 | 10545 | 15633 |
| 1.533 | 4276.4 | 4889.9 | 6205.3 | 7103.1 | 10642 | 15833 |
| 1.5414 | 4503.3 | 5326.8 | 6357.1 | 7422.7 | 10685 | 16014 |
| 1.5498 | 3809.7 | 4717.7 | 5822.5 | 6775 | 10269 | 15376 |
| 1.5582 | 3939.3 | 4738.6 | 5805.7 | 6908.9 | 10236 | 15779 |
| 1.5666 | 4008.1 | 4595.1 | 5765 | 6636.2 | 10324 | 15772 |
| 1.575 | 3701.4 | 4393.2 | 5543 | 6573.4 | 10230 | 15743 |
| 1.5834 | 3406.3 | 4266.2 | 5339.3 | 6440.4 | 9697.8 | 15489 |
| 1.5918 | 3540.5 | 3990.7 | 5073.1 | 6234 | 9543.8 | 15665 |
| 1.6002 | 3033 | 3826 | 4967.3 | 6049.1 | 9329.3 | 15244 |
| 1.6086 | 3187.6 | 4088.8 | 5177.2 | 6076.6 | 9389.7 | 15194 |
| 1.6169 | 2967.6 | 3952.6 | 4894.2 | 5919.5 | 9232.3 | 15149 |
| 1.6253 | 3331.1 | 4294.7 | 5332.1 | 6157.4 | 9431.5 | 15606 |
| 1.6337 | 3383.7 | 4081.9 | 5442.9 | 6167.7 | 9471 | 15489 |
| 1.6421 | 3277.7 | 4166 | 5502 | 6316.6 | 9352 | 15393 |
| 1.6505 | 3166.8 | 3973.3 | 5225.2 | 6090.8 | 9168.5 | 15059 |
| 1.6589 | 3182.1 | 4089.2 | 5258.1 | 6000.8 | 9107.7 | 15029 |
| 1.6673 | 3238.5 | 4121.6 | 5208.5 | 6143 | 9118.5 | 14847 |
| 1.6757 | 2910.3 | 3751.5 | 4882.7 | 5976.2 | 8738.6 | 14345 |
| 1.6841 | 3063.8 | 4006.5 | 5112 | 6103.2 | 9070.4 | 14301 |
| 1.6925 | 2935.3 | 3931.9 | 4889.2 | 6088.3 | 9050.2 | 14219 |
| 1.7009 | 3256.7 | 4122.6 | 5190.7 | 6361.7 | 9452.4 | 13996 |
| 1.7093 | 3186.2 | 3976.2 | 5111.6 | 6164.9 | 9159.6 | 13625 |
| 1.7177 | 3341.7 | 4291.1 | 5453.9 | 6482.9 | 9571.4 | 13831 |
| 1.726 | 3306.5 | 4166.6 | 5231.6 | 6338.3 | 9517.8 | 13924 |
| 1.7344 | 2811.7 | 3787.8 | 4990.9 | 6084.3 | 9027.9 | 13493 |
| 1.7428 | 3101.5 | 4978.6 | 4927.6 | 6501.9 | 9289.9 | 13266 |
| 1.7512 | 3190.1 | 4016.5 | 4976.1 | 6509.3 | 9150.8 | 13364 |
| 1.7596 | 3040.7 | 3805.8 | 4871.3 | 6535.4 | 8929.9 | 13294 |
| 1.768 | 3198.1 | 3856.3 | 4902.5 | 6387.6 | 8988.2 | 13229 |
| 1.7764 | 3235.7 | 4009.5 | 5063.3 | 6438.1 | 8803.4 | 14105 |
| 1.7848 | 3421.3 | 4204.6 | 5289 | 6713 | 9164.5 | 13867 |
| 1.7932 | 3128 | 4191.2 | 4940.4 | 6386 | 8676.7 | 13615 |
| 1.8016 | 3365.8 | 4219.1 | 5284 | 6306.7 | 8811.6 | 14015 |
| 1.81 | 3378.8 | 4223.9 | 5193.2 | 6408.7 | 8643.8 | 13971 |
| 1.8184 | 3549.9 | 4319.4 | 5571.6 | 6411.6 | 8555.4 | 13974 |
| 1.8268 | 3583.2 | 4410.7 | 5651.5 | 6350.8 | 8598.1 | 13855 |
| 1.8351 | 3358.5 | 4156.3 | 5404.2 | 6030.3 | 8569 | 13384 |
| 1.8435 | 3482.1 | 4222.1 | 5365.8 | 6033.1 | 8707.4 | 13225 |
| 1.8519 | 3442.7 | 4218.1 | 5179.7 | 5974.4 | 8747.7 | 12730 |
| 1.8603 | 3409.5 | 4282.7 | 5050 | 6013.8 | 8787 | 12267 |
| 1.8687 | 3507.3 | 4411 | 5156.1 | 6165.5 | 8815 | 12057 |
| 1.8771 | 3470 | 4289.6 | 5159.5 | 6082.3 | 8688.9 | 11704 |
| 1.8855 | 3299.1 | 4034.1 | 4768.5 | 5786.2 | 8557.3 | 11233 |
| 1.8939 | 3118 | 3869.1 | 4607.4 | 5604.8 | 8262.7 | 11101 |
| 1.9023 | 3048 | 3748.6 | 4574.5 | 5327.3 | 8317.6 | 10923 |
| 1.9107 | 3024.7 | 3720.3 | 4575.1 | 5394.5 | 8211.9 | 10915 |
| 1.9191 | 2802.5 | 3486.8 | 4307.6 | 5114 | 7901.2 | 10632 |
| 1.9275 | 2640.9 | 3314.6 | 4117.6 | 4809.5 | 7653.2 | 10400 |
| 1.9359 | 2639.9 | 3262.9 | 4078.2 | 4683.5 | 7484.5 | 10327 |
| 1.9442 | 2496.6 | 2935.5 | 3696.5 | 4349.8 | 7020 | 9949.1 |
| 1.9526 | 2408.1 | 3088.9 | 3708.7 | 4231.9 | 6853.1 | 9829.5 |
| 1.961 | 2236.9 | 2891 | 3554.4 | 3869.5 | 6449.7 | 9422.4 |
| 1.9694 | 2085.2 | 2662.7 | 3340.2 | 3652.9 | 6122.9 | 9065.4 |
| 1.9778 | 1973.3 | 2527.8 | 3135.9 | 3540.6 | 5858.6 | 8730.8 |
| 1.9862 | 1887.1 | 2503.4 | 2993.1 | 3352.9 | 5541.1 | 8282.2 |
| 1.9946 | 1797.9 | 2347 | 2978.6 | 3210.4 | 5325.3 | 7913.1 |
| 2.003 | 1758.4 | 2252.4 | 2781.4 | 3095.1 | 5083 | 7643 |
| 2.0114 | 1655.9 | 2103.8 | 2623.4 | 2939.8 | 4822.2 | 7361.4 |
| 2.0198 | 1510.5 | 1928.1 | 2560.3 | 2760.4 | 4557.9 | 7068.5 |
| 2.0282 | 1508.4 | 1937.6 | 2523.2 | 2678.7 | 4325.8 | 6889.6 |
| 2.0366 | 1486.5 | 1860 | 2308.5 | 2545.5 | 4148.2 | 6536 |
| 2.045 | 1411.9 | 1799.4 | 2228.9 | 2434.9 | 4013.1 | 6215.8 |
| 2.0533 | 1322.6 | 1759.3 | 2250.3 | 2363 | 3899.3 | 5954.5 |
| 2.0617 | 1352.3 | 1696.5 | 2114 | 2346 | 3750.4 | 5727.8 |
| 2.0701 | 1227.4 | 1586.5 | 1997.6 | 2137.1 | 3456.2 | 5457.4 |
| 2.0785 | 1135.9 | 1458.8 | 1872.5 | 1986.4 | 3229 | 5231 |
| 2.0869 | 1124.2 | 1329.1 | 1735.9 | 1953.7 | 3122.1 | 5065.7 |
| 2.0953 | 1077.4 | 1341.5 | 1687.8 | 1862.6 | 3028.2 | 4826.3 |
| 2.1037 | 1067.8 | 1343.5 | 1686 | 1869.4 | 2992.6 | 4655.4 |
| 2.1121 | 1091.6 | 1327.9 | 1696.6 | 1908.6 | 2954 | 4529.2 |
| 2.1205 | 1162.6 | 1268.6 | 1658.9 | 1869.1 | 2785.4 | 4426.5 |
| 2.1289 | 945.26 | 1100.9 | 1468.2 | 1632.5 | 2539.4 | 4212.6 |
| 2.1373 | 775.64 | 995.41 | 1341.5 | 1462.8 | 2409.8 | 4062.4 |
| 2.1457 | 890.61 | 1092.1 | 1449.6 | 1561.4 | 2579.6 | 4119 |
| 2.1541 | 797.74 | 1046.9 | 1424.5 | 1536.4 | 2562.7 | 4004.6 |
| 2.1624 | 735.74 | 1010.9 | 1385.6 | 1526.1 | 2499.7 | 3896 |
| 2.1708 | 806.66 | 1043.3 | 1369.1 | 1588.2 | 2417.9 | 3859.5 |
| 2.1792 | 765.7 | 1027 | 1341.9 | 1578.7 | 2365.5 | 3713.1 |
| 2.1876 | 768.54 | 1056.2 | 1341.2 | 1565.3 | 2383.6 | 3643.6 |
| 2.196 | 918.86 | 1208.7 | 1404.5 | 1574.6 | 2546.2 | 3793.1 |
| 2.2044 | 925.74 | 1211.1 | 1414.7 | 1569.7 | 2544.6 | 3744.8 |
| 2.2128 | 903.77 | 1175.7 | 1401.3 | 1572.5 | 2491.1 | 3664.1 |
| 2.2212 | 947.22 | 1177 | 1359.1 | 1625.7 | 2434.3 | 3702.2 |
| 2.2296 | 934.79 | 1169.6 | 1377.5 | 1627.8 | 2447.1 | 3728.4 |
| 2.238 | 887.98 | 1181 | 1432.2 | 1598.2 | 2501.7 | 3745.4 |
| 2.2464 | 853.52 | 1121.4 | 1371.8 | 1568.5 | 2420.8 | 3549.9 |
| 2.2548 | 842.09 | 1145.3 | 1343.1 | 1550.4 | 2360.2 | 3459.5 |
| 2.2632 | 865.89 | 1087.3 | 1362.9 | 1550 | 2330.3 | 3529.4 |
| 2.2715 | 807.24 | 1044.4 | 1317.7 | 1497.6 | 2277.5 | 3479.3 |
| 2.2799 | 724.58 | 960.01 | 1254.8 | 1435.3 | 2223.6 | 3390.7 |
| 2.2883 | 669.63 | 944.27 | 1227.1 | 1444.7 | 2233.6 | 3308.6 |
| 2.2967 | 689.54 | 998.93 | 1255.5 | 1498.3 | 2288.6 | 3315.4 |
| 2.3051 | 817.14 | 1155.3 | 1364.6 | 1616.2 | 2409 | 3449.9 |
| 2.3135 | 899.46 | 1168.1 | 1393.1 | 1618.4 | 2402.3 | 3476.4 |
| 2.3219 | 961.96 | 1121.3 | 1387.8 | 1572.7 | 2343 | 3457.7 |
| 2.3303 | 999.82 | 1195.5 | 1452.3 | 1596.3 | 2391.6 | 3466.5 |
| 2.3387 | 996.32 | 1226.1 | 1472.4 | 1606.6 | 2400.5 | 3458.8 |
| 2.3471 | 861.59 | 1108.9 | 1345.4 | 1569.2 | 2274.3 | 3396.1 |
| 2.3555 | 825.14 | 1048.6 | 1295.7 | 1549.8 | 2232.5 | 3349.3 |
| 2.3639 | 898.49 | 1051.6 | 1332.2 | 1550.3 | 2284.7 | 3320 |
| 2.3723 | 912.83 | 1076.1 | 1371 | 1588.4 | 2340.6 | 3335.3 |
| 2.3807 | 899.4 | 1110.2 | 1410.5 | 1643.6 | 2397.5 | 3371.2 |
| 2.389 | 883.9 | 1122.3 | 1420.2 | 1589.2 | 2329.7 | 3328.8 |
| 2.3974 | 866.98 | 1128.3 | 1421.2 | 1519.4 | 2243.6 | 3272.3 |
| 2.4058 | 800.92 | 1042.3 | 1278.7 | 1478.4 | 2155.3 | 3129.8 |
| 2.4142 | 752.16 | 977.2 | 1170 | 1445.3 | 2092.1 | 3031.8 |
| 2.4226 | 783.69 | 1008.3 | 1217.8 | 1448 | 2146 | 3142.6 |
| 2.431 | 776.01 | 1002.4 | 1218.4 | 1432.8 | 2144.5 | 3165.1 |
| 2.4394 | 671.62 | 905.22 | 1103.2 | 1373 | 2006.4 | 2971.2 |
| 2.4478 | 632 | 872.44 | 1080.6 | 1327 | 1948.4 | 2898.4 |
| 2.4562 | 697.05 | 943.85 | 1208.1 | 1303 | 2019.5 | 3021.1 |
| 2.4646 | 721.02 | 968.51 | 1242.6 | 1285.9 | 2047.4 | 3063.9 |
| 2.473 | 694.43 | 935.63 | 1162.6 | 1277.2 | 2022 | 3008.4 |
| 2.4814 | 665.62 | 898.41 | 1105.7 | 1257.4 | 1976.5 | 2917.2 |
| 2.4897 | 634.46 | 856.6 | 1072.9 | 1225.9 | 1910 | 2788.4 |
| 2.4981 | 684.17 | 863.59 | 1104.2 | 1256.2 | 1907.9 | 2775.2 |
| 2.5065 | 813.95 | 918.89 | 1198.9 | 1347.7 | 1969.5 | 2876.6 |
| 2.5149 | 817.83 | 915.77 | 1209 | 1354.1 | 1949.1 | 2880.3 |
| 2.5233 | 690.74 | 851.87 | 1131.1 | 1271.9 | 1843.5 | 2782.4 |
| 2.5317 | 635.73 | 837.63 | 1100 | 1253.2 | 1807.8 | 2721.2 |
| 2.5401 | 668.46 | 883.97 | 1126.1 | 1311.7 | 1857.6 | 2704.4 |
| 2.5485 | 687.06 | 891.22 | 1121.5 | 1337.9 | 1886 | 2685.2 |
| 2.5569 | 683.08 | 835.46 | 1067.5 | 1312.1 | 1880.2 | 2662 |
| 2.5653 | 701.18 | 825.92 | 1044.3 | 1297.7 | 1875.5 | 2650.5 |
| 2.5737 | 775.18 | 931.88 | 1097.4 | 1311.4 | 1873.4 | 2668.3 |
| 2.5821 | 811.72 | 991.46 | 1120 | 1296.7 | 1848.3 | 2658.2 |
| 2.5905 | 652.49 | 809.92 | 982.42 | 1130.2 | 1699.6 | 2499.7 |
